# Zinc Chelation Specifically Inhibits Early Stages of Dengue Virus Replication by Activation of NF-κB and Induction of Antiviral Response in Epithelial Cells

**DOI:** 10.3389/fimmu.2019.02347

**Published:** 2019-10-01

**Authors:** Meenakshi Kar, Naseem Ahmed Khan, Aleksha Panwar, Sachendra S. Bais, Soumen Basak, Renu Goel, Shailaja Sopory, Guruprasad R. Medigeshi

**Affiliations:** ^1^Clinical and Cellular Virology Lab, Translational Health Science and Technology Institute, Faridabad, India; ^2^Systems Immunology Laboratory, National Institute of Immunology, New Delhi, India; ^3^Drug Discovery Research Centre, Translational Health Science and Technology Institute, Faridabad, India; ^4^Pediatric Biology Centre, Translational Health Science and Technology Institute, Faridabad, India

**Keywords:** dengue virus, zinc, rotavirus, epithelial cells, NF-kappaB

## Abstract

Zinc is an essential micronutrient which regulates diverse physiological functions and has been shown to play a crucial role in viral infections. Zinc has a necessary role in the replication of many viruses, however, antiviral action of zinc has also been demonstrated in *in vitro* infection models most likely through induction of host antiviral responses. Therefore, depending on the host machinery that the virus employs at different stages of infection, zinc may either facilitate, or inhibit virus infection. In this study, we show that zinc plays divergent roles in rotavirus and dengue virus infections in epithelial cells. Dengue virus infection did not perturb the epithelial barrier functions despite the release of virus from the basolateral surface whereas rotavirus infection led to disruption of epithelial junctions. In rotavirus infection, zinc supplementation post-infection did not block barrier disruption suggesting that zinc does not affect rotavirus life-cycle or protects epithelial barriers post-infection suggesting the involvement of cellular pathways in the beneficial effect of zinc supplementation in enteric infections. Zinc depletion by N,N,N',N'-tetrakis(2-pyridinylmethyl)-1,2-ethanediamine (TPEN) inhibited dengue virus and Japanese encephalitis virus (JEV) infection but had no effect on rotavirus. Time-of-addition experiments suggested that zinc chelation affected both early and late stages of dengue virus infectious cycle and zinc chelation abrogated dengue virus RNA replication. We show that transient zinc chelation induces ER stress and antiviral response by activating NF-kappaB leading to induction of interferon signaling. These results suggest that modulation of zinc homeostasis during virus infection could be a component of host antiviral response and altering zinc homeostasis may act as a potent antiviral strategy against flaviviruses.

## Introduction

Zinc is the most abundant micronutrient after iron and zinc deficiency rates in developing countries range from 20 to 30%. Zinc is known to regulate the functions of about 10% of the human proteome and a large number of physiological processes that are zinc dependent have been identified and characterized under conditions of zinc deficiency and supplementation. Some of the predominant zinc-dependent functions such as immune response, metabolism, nucleic acid synthesis and repair, apoptosis and redox homeostasis act as important determinants of host-pathogen interactions. As zinc homeostasis is closely linked to the normal functioning of both prokaryotic and eukaryotic cells, many pathogens are directly, or indirectly affected by perturbations in zinc homeostasis. Zinc finger proteins have been shown to play both proviral and antiviral roles in a number of studies with different viruses (1–3). Previous reports have also shown divergent effects of Zn^2+^ on virus replication. Zinc inhibited RNA polymerase activity of a number of viruses including coronavirus, arterivirus, rhinovirus, and hepatitis C virus (4–9). Dengue virus (DENV), a mosquito-borne, positive-strand RNA virus from the family *Flaviviridae*, has emerged as one of the major public health concerns in India and recent estimates suggest that over 60 million people globally get infected with DENV every year (10). The crystal structures of NS5 protein of DENV and West Nile virus have identified zinc binding site in RdRp domain and propose an important structural role for zinc ions in polymerase activity (11, 12). Therefore, zinc is likely to play a prominent role in the polymerase activity of DENV. One of the prominent physiological functions of zinc is in the regulation of permeability barrier functions in epithelial and endothelial cells. Although many previous reports have focused on the pathogenesis of dengue using vascular endothelial cells, the effect of dengue virus infection on epithelial barrier functions has not been characterized. Epithelial cells play a major role in dengue transmission by mosquitos and animal studies indicate that epithelial cells are active sites of virus infection and contribute to pathogenesis (13, 14). Considering the important role of zinc in epithelial barrier functions, we were interested in investigating the effect of virus infection on epithelial barrier functions and to test whether zinc has a functional role in epithelial cells infected with dengue virus. We show that, similar to mid gut epithelial cells in mosquitos, dengue virus infected human intestinal epithelial cells, and was capable of exiting infected cells from both apical and basolateral surface without disrupting cellular junctions or affecting barrier functions unlike rotavirus infection which disrupted the epithelial barrier. Addition of ZnSO_4_ in the culture medium after virus adsorption had no effect on viral titres or barrier functions. We utilized N,N,N',N'-tetrakis(2-pyridinylmethyl)-1,2-ethanediamine (TPEN), a zinc-specific chelator, to mimic acute zinc-deficiency in cell culture models of infection and investigated the effect of zinc depletion on DENV, Japanese encephalitis virus (JEV), and rotavirus (RV) infections. In this study, zinc chelation showed an inhibitory effect on DENV and JEV replication but had no effect on RV infection. We further probed the mechanism of action of TPEN and showed zinc chelation affect early stages of dengue life-cycle by inhibiting DENV RNA replication. By RNA seq analysis we observed that zinc chelation leads to induction of ER stress and heat shock proteins, activation of NF-κB leading to upregulation of a subset of type I interferon-dependent antiviral response which specifically blocks DENV replication. Thus, our study provides information on the role of zinc in flavivirus infection and demonstrates that alteration of zinc homeostasis leads to induction of an antiviral response involving ER stress and NF-κB that specifically affects flaviviruses.

## Materials and Methods

### Cells and Viruses

Caco-2, A549, Huh-7, BHK-21, C6/36, HEp-2, PS, and MA104 cells were cultured as described previously (15–17). Virus strains used in the study and infection procedures has been described before (15–17). DENV-2 New Guinea C strain was a kind gift from Dr. Navin Khanna. Rotavirus was activated using 10 μg/mL trypsin (Worthington Biochemical Corporation) for 1 h at 37°C. Caco-2 cells were infected with activated virus at 0.5 MOI for 1 h in serum-free medium. After adsorption cells were washed twice with PBS and cells were grown in serum-free medium containing trypsin at 0.5 μg/mL. Throughout this study, we have used two epithelial cell lines, Caco-2 and A549 cells as both these cells support DENV infection. In all the experiments, Caco-2 cells were infected with DENV at 10 MOI and A549 at 5 MOI. Most of the experiments have been performed in both the cell lines and we have obtained similar results. Plaque assay for DENV, JEV, and RV was set up in BHK-21, PS, and MA 104 cells respectively as described previously (15–17).

### Treatment of Cells

Cells were treated with 0.5 μM *N, N, N*′, *N*′-tetrakis(2-pyridinylmethyl)-1,2-ethanediamine (TPEN) (Sigma) in serum-free media for chelation of intracellular zinc. Zinc levels were measured by flow cytometry as described in the labile zinc measurement section below. To study the effect of TPEN on virus infection, 0.5 μM TPEN was added in serum-free media after infection. At indicated time points, supernatant was collected for estimating virus titer by plaque assay and cells were collected for western blotting or quantitative real-time PCR (qRT-PCR) as described in the following sections. For time-of-addition experiments, Caco-2 cells infected with DENV were treated with TPEN at 8, 16, and 24 h pi. Supernatants were collected at 16 h post-addition of TPEN for measuring viral titers. Cells were used for RNA isolation and RT-PCR as described in the later sections. For rescue experiments, cells were treated with DMSO or TPEN for 4 h and medium containing TPEN was supplemented with 10 μM of ZnSO_4_/MnCl_2_/MgCl_2_/CuCl_2_/FeSO_4._ For pre-treatment experiments, cells were treated with DMSO or 0.5 μM TPEN for 4 h. Cells were washed with PBS and infected with 10 MOI of DENV-2 and cultured for 24 h in DMEM containing 2% FBS without TPEN. For labile zinc recovery experiments, cells were treated with TPEN for 4 h, washed twice with PBS and cultured in serum-free medium for indicated periods and labile zinc pools were measured by confocal microscopy or flow cytometry. For Salubrinal treatment, cells were infected with DENV and, 1 h pi, culture medium was supplemented with 50 μM Salubriinal along with DMSO or TPEN. Viral titres were estimated at 24 h pi.

### Labile Zinc Measurement by Flow Cytometry

Cells were washed once with PBS after treatment or infection following which they were detached using trypsin (80–120 μL) (Gibco) and collected by adding defined trypsin inhibitor (Gibco). Cells were resuspended in DMEM without phenol red (Gibco) supplemented with 2 mM L-glutamine (Gibco) (staining media). Cells were stained using either 5 μM Fluozin-3-AM (Molecular Probes) or 2.5 μM ZinPyr-1 (ZP-1) (Santa Cruz) in the staining media. For ZP-1 staining, medium containing 1 mM EDTA was added to chelate any extracellular zinc during staining. Cells were incubated for 30 min at 37°C in CO_2_ incubator and mixed every 10 min. For assessing cell viability, fixable viability stain eFluor780 (Becton Dickinson Biosciences) was added to the cells at 1:500 dilution prepared in the staining medium and incubated for further 10 min at 37°C in the CO_2_ incubator. Cells were washed using FACS buffer (PBS containing 0.25% FBS) and acquired in FACS Canto II (Becton Dickinson). The amount of labile zinc present in live cells was presented as the mean fluorescence intensity of Fluozin-3-AM and ZP-1. Images were quantitated using cellSens Software (Olympus).

### Quantitative RT-PCR Assay

Caco-2 cells were infected with DENV-2 at a MOI of 10 for 1 h. Similarly, A549 cells were infected with DENV-2 at a MOI of 5. After 1 h of virus adsorption cells were washed twice with PBS and serum-free DMEM supplemented with 0.5 μM TPEN was added. At indicated time points, supernatant was collected for estimating viral titres by plaque assay and cells were harvested for positive and negative strand detection PCR as described previously (18). Briefly, RNA was isolated using RNAiso Plus (Takara) as per manufacturer's instructions and reverse transcribed using forward or reverse primer. RT product was further amplified using primer and probe mix using TaqMan RNA-to-Ct one step kit (Applied Biosystems) as described previously (19). GAPDH primer probe mix (Applied Biosystems) was used as housekeeping control and used for normalizing dengue genome levels. Data was analyzed using ΔΔCt method. Viral entry experiments were performed by infecting Caco-2 cells with 10 MOI of DENV-2. One hour after virus adsorption, cells were collected by trypsinization and trypsin was inactivated by resuspending cells in complete medium. Cells were washed twice with PBS and total RNA was isolated and internalized DENV genome levels were determined as described above. For interferon pathway, RNA was isolated from cells at 4 and 8 h post-TPEN treatment and the indicated genes were quantitated by SyBR green chemistry using GAPDH as housekeeping control gene for normalization. The list of primers used is provided in Supplementary Information (Supplementary Table 1). Rotavirus genome levels were quantitated as described previously (20).

### Confocal Microscopy and Barrier Studies

Caco-2 and A549 cells were seeded at 30,000 cells per well in 3 μm pore size polycarbonate membranes for 4 days. Media was changed every alternate day and trans-electrical epithelial resistance (TEER) was monitored using a chopstick electrode. At day four, cells were washed with cold PBS and fixed in ice-cold methanol for 20 min at −20°C. Cells were washed twice using PBS followed by blocking using 0.2% BSA in IMF buffer for 5–10 min at room temperature (RT). Cells were incubated with antibodies against β-catenin (Sigma) and occludin (Invitrogen) in IMF buffer (20 mM HEPES pH 7.5, 0.1% Triton X-100, 150 mM NaCl, 5 mM EDTA, 0.02 % sodium azide) for 1 h at RT followed by washing and incubation with Alexa flour 568 tagged secondary antibodies (Molecular probes) for 30 min at RT in dark. Cells were washed with IMF buffer three times and stained with 4′,6-diamidino-2-phenylindole (DAPI) (Molecular probes) at 1:10,000 dilution for 10 min. Cells were washed with PBS, mounted using antifade solution (Molecular probes) and imaged using FV1000 fluorescence microscope (Olympus).

To assess the effect of zinc on epithelial barrier, Caco-2 and A549 cells were grown for 4 days in transwells. Cells were washed with PBS and 2% medium containing ZnSO_4_ was added apically, basolaterally, or both sides of the transwells. TEER was monitored and cells were used for measuring zinc uptake and viability by flow cytometry as explained in labile zinc measurement section.

To assess effect of DENV and RV infection on epithelial cells barrier, Caco-2 cells were grown in transwells for 4 days and infected with DENV-2 and RV at 10 and 0.5 MOI, respectively as mentioned above. TEER was monitored till 72 h pi for DENV infection and 16 h pi for RV infection. Supernatants were collected from both apical and basolateral surface for measuring viral titres by plaque assay. Effect of zinc in rescuing RV induced barrier damage was assessed by adding 50 μM ZnSO_4_ in the media of infected cells and measuring TEER and viral titres.

### Cytotoxicity and Cell Proliferation Assays

Cytotoxicity was assessed by CytoTox 96 Non-Radioactive Cytotoxicity Assay kit (Promega) which measures lactate dehydrogenase (LDH) activity in the culture supernatants. As a positive control for cytotoxicity, cells were lysed in 0.1% Triton-X-100 and the cell lysate was used for LDH measurement. The amount of LDH activity in the detergent-treated sample was considered as 100% and the sample's cytotoxicity was measured relative to the detergent control. Cell proliferation was measured using CellTiter 96® AQ_ueous_ One Solution Cell Proliferation Assay (Promega) as per the manufacturer's instructions.

### Electrophoresis Mobility Shift Assays (EMSA)

Caco-2 cells were treated with DMSO or TPEN as described above for 4 and 8 h. Nuclear extracts were prepared and processed for EMSA as described previously (21). Briefly, nuclear extracts (NE) were prepared after treating Caco-2 cells with 0.5 μM TPEN for indicated periods and normalized for protein concentration. Thereafter, NE were incubated with NF-κB or Oct1 specific probes and then were resolved on native PAGE gel. The gel images were acquired using PhosphorImager (GE, Amersham, UK) and quantified in ImageQuant 5.2.

### RNA Seq Analysis

#### Sample Preparation

Caco-2 cells were seeded in a 24-well plate at the cell density of 100,000 cells per well. On day 2 post-seeding, cells were washed twice with PBS and serum-free DMEM containing DMSO and TPEN (0.5 μM) were added to respective wells and cultured for 4 h. After 4 h of treatment, media was removed and cells were washed twice with PBS. Cells were collected in 300 μl RNA lysis buffer (RNA prep kit-Zymo-R1051). RNA was prepared according to the manufacturer's instructions and eluted in 20 μl nuclease-free water. One microgram of total RNA was used for the construction of sequencing libraries using Illumina TruSeq RNA Sample Prep Kit (Cat#FC-122-1001).

For library construction, total RNA is extracted from a sample. After performing quality control (QC), passed sample is proceeded with the library construction. The extracted RNA with an RNA integrity number (RIN) of 7.0 was used for mRNA purification. mRNA was purified using oligo-dT beads (TruSeq RNA Sample Preparation Kit, Illumina) taking 1 μg of intact total RNA. The purified mRNA was fragmented at 90°C in the presence of divalent cations. The fragments were reverse transcribed using random hexamers and Superscript II Reverse Transcriptase (Life Technologies). Second strand cDNA was synthesized on the first strand template using RNaseH and DNA polymerase I. The cDNAs so obtained were cleaned using Beckman Coulter Agencourt Ampure XP SPRI beads.

#### Library Construction

The sequencing library is prepared by random fragmentation of the cDNA sample, followed by 5′ and 3′ adapter ligation, after end-repair and the addition of an “A” base and SPRI cleanup. The prepared cDNA library was amplified using PCR for the enrichment of the adapter-ligated fragments. Alternatively, “tagmentation” combines the fragmentation and ligation reactions into a single step that greatly increases the efficiency of the library preparation process. The individual libraries were quantified using a NanoDrop spectrophotometer (Thermo Scientific) and validated for quality with a Bioanalyzer (Agilent Technologies). Adapter-ligated fragments are then PCR amplified and gel purified.

#### Sequencing

For cluster generation, the library is loaded into a flow cell where fragments are captured on a lawn of surface-bound oligos complementary to the library adapters. Each fragment is then amplified into distinct, clonal clusters through bridge amplification. When cluster generation is complete, the templates are ready for sequencing. Illumina SBS technology utilizes a proprietary reversible terminator-based method that detects single bases as they are incorporated into DNA template strands. As all 4 reversible, terminator-bound dNTPs are present during each sequencing cycle, natural competition minimizes incorporation bias and greatly reduces raw error rates compared to other technologies. The result is highly accurate base-by-base sequencing that virtually eliminates sequence-context-specific errors, even within repetitive sequence regions and homopolymers.

#### RNA Seq Data Analysis

Stringent quality control of Paired End sequence reads of all the samples was done using NGSQCTool kit (22). Paired end sequence reads with Phred score >Q30 was taken for further analysis. NCBI *Homosapien* Hg38 genome file was used for read alignment and identification of transcripts. TopHat pipeline (23) was used for alignment and Cufflink and Cuffdiff pipeline (24) was used for identification of transcript coding regions followed by quantitation and annotation using default parameters. Unsupervised hierarchical clustering of differentially expressed genes was done using Cluster 3.0 (25) and visualized using Java Tree View (26). Gene ontologies and pathways that harbor expressed transcripts were identified using DAVID Functional Annotation Tool [DAVID Bioinformatics Resources 6.8, NIAID/NIH]. Differentially expressed transcripts between Control and Treated samples were identified by CuffDiff data analysis pipeline using a fold-change threshold of absolute fold-change ≥1.5 and a statistically significant Student's *t*-test *P* value threshold adjusted for false discovery rate of <0.001. Statistically significantly enriched functional classes with a *P* value adjusted for false discovery rate of <0.05 derived using the hypergeometric distribution test corresponding to differentially expressed genes were determined using Student's *t*-test with Benjamini Hocheberg FDR test. The heat map for display expression pattern were obtained using Cluster 3.0 for normalizing and hierarchical clustering with average linkage based on Pearson coefficients, followed by Java Tree-View 1.1 program for visualizing the analyzing datasets (25, 26).

Furthermore, analysis was performed to identify the significant pathways, functions, and networks associated with significantly differentially expressed gene transcripts by using ingenuity pathway analysis (IPA) software (Ingenuity Systems, Redwood City, CA, www.qiagen.com/ingenuity). Differentially expressed (upregulated or downregulated) gene transcripts that showed a minimum of 2 fold change in DMSO samples as compared to that of TPEN treatment was imported to IPA for the core analysis. These differentially regulated genes are known as focus genes in IPA. A *p*-value was calculated using right-tailed Fisher's exact test to explain the significance of the pathways related to our genes list and it was set at <0.05 (or score > 1.3 score = –lop *P*). Additionally, all dysregulated transcripts were analyzed for the prediction of activation or inhibition of canonical pathway based on z-score. IPA automatically calculates the z-score in the range of ~ −2 < z >2 based on differentially expressed genes from our dataset with the information stored in IPA knowledge database. Positive and negative z-score suggests the activation and inhibition of the pathway, respectively. The pathways are displayed graphically as a collection of nodes i.e., gene transcripts and edges-the biological relationships between the nodes. Different shades of red and green nodes reflect the relative fold change of gene transcripts. IPA algorithm has given white nodes to the genes not present in our list of differentially expressed genes. Solid and dotted lines indicate direct and indirect interaction or regulation, respectively. Each line is has at least one reference from the literature.

### Data Analysis

Data was analyzed and charts were prepared using GraphPad Prism software. All experiments were performed with two or more replicates and graphs have been prepared representing data from at least two independent experiments with *n* ≥ 6. Error bars represent mean ± SD. Statistical significance was estimated by *t*-test (unpaired, non-parametric) using Mann-Whitney test.

## Results

### Epithelial Barrier Functions Are Regulated by Zinc Homeostasis

**Figure 1:**
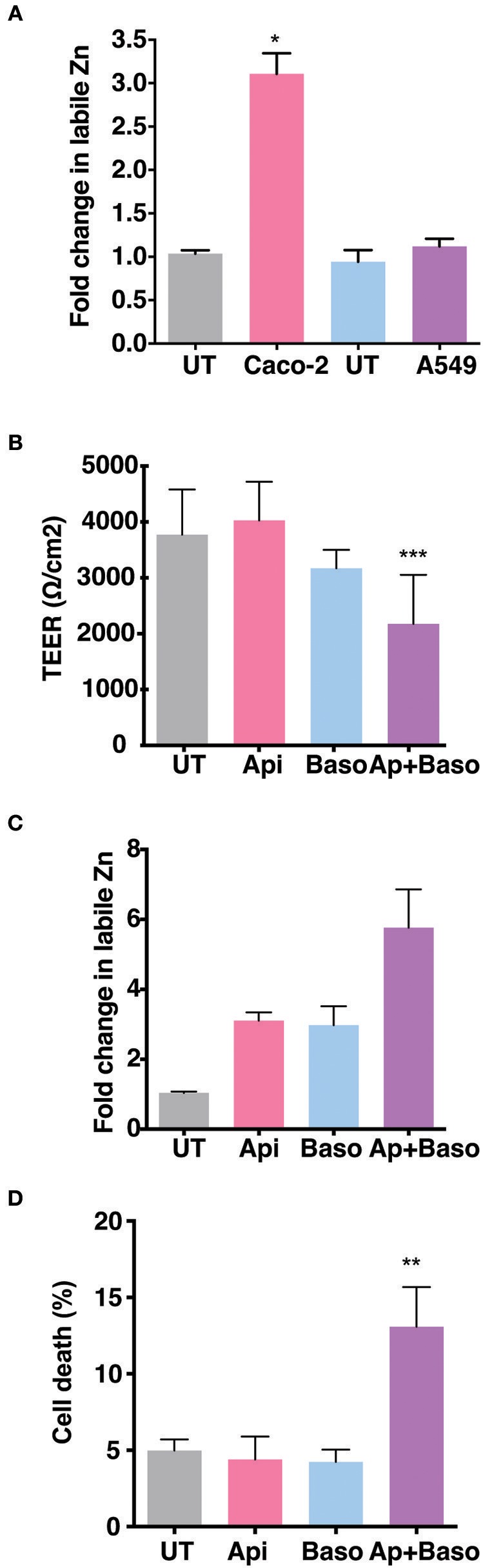
Role of Zn in barrier functions in Caco-2 and A549 cells. Caco-2 and A549 cells were seeded at 30,000 cells per well and cultured in 3.0 μm transwell inserts for 4 days. **(A)** Comparison of zinc uptake in Caco-2 and A549 cells treated apically with 100 μM ZnSO_4_ in 2% media for 24 h. **(B)** TEER of Caco-2 cells grown for 7 days treated with ZnSO_4_ at 100 μM by apical, basolateral or apical + basolateral regions in 2% DMEM for 24 h. **(C)** Corresponding fold change in zinc levels in Caco-2 cells measured by FLZ-3AM staining at 24 h post treatment. **(D)** Percentage of dead cells in apical, basolateral, and apical + basolateral ZnSO_4_ treated Caco-2 cells at 24 h measured by live dead stain using flow cytometry. Data are from at least two independent experiments and represent mean ± SD. *p < 0.05, **p < 0.01, ***p < 0.001.

The role of Zinc on epithelial and endothelial permeability barrier functions has been demonstrated by a number of *in vitro* and *in vivo* studies. We were interested to test the effect of zinc supplementation in the context of permeability barrier functions in cells infected with viruses. We first examined the barrier properties of two epithelial cell lines Caco-2 (colon) and A549 (lung) by growing these cells on transwell inserts for 4 days and measuring the trans-epithelial electrical resistance (TEER) every day. Caco-2 cells have been reported to have higher expression of tight junction proteins and undergo differentiation whereas A549 cells have lower TEER values and do not undergo differentiation (27–30). As expected, the basal levels of TEER was about 200-fold lower in A549 cells as compared to Caco-2 on day 2, however, both cell lines showed an increase in the TEER values with time in culture and started to plateau by day 4 (Supplementary Figures 1A,B). We stained these transwells for occludin and β-catenin, a marker for tight junction and adherens junction, respectively. A549 cells showed a diffused and weak occludin staining while β-catenin staining showed a typical adherens junction pattern. In Caco-2 cells, both occludin and β-catenin showed a clear and intense tight and adherens junctional staining, respectively (Supplementary Figure 1C). To further determine the capacity to uptake Zn by these cells, we added ZnSO_4_ in the apical medium and measured labile zinc levels after 24 h in both the cells by flow cytometry by zinc fluorophore, fluozin-3AM. Caco-2 cells showed a 3-fold increase in labile zinc levels under these conditions whereas labile zinc levels was unchanged in A549 cells (Figure 1A). These results suggest that A549 cells have very poor zinc uptake capacity as compared to Caco-2 cells. Therefore, all further experiments were performed in Caco-2 cells. We next measured the effect of zinc supplementation on barrier integrity in Caco-2 cells. Cells were grown for 4 days and Zn was added either to the apical medium or in the basolateral medium or in both the apical and basolateral chambers for 24 h. Zn supplementation had no effect in Caco-2 when added only in the apical or basolateral medium. However, when both apical and basolateral medium was supplemented with Zn, TEER values decreased significantly (Figure 1B). We next verified Zn uptake under these conditions by measuring labile Zn levels using fluozin-3AM, a cell permeable zinc fluorophore, by flow cytometry and observed about 2.5-fold uptake when Zn was added either on the apical or basolateral side and 5.5-fold increase in labile Zn levels when Zn was added in both apical and basolateral media (Figure 1C). To test if this increase in intracellular zinc leads to compromise in cell viability, we assessed cell viability by live-dead stain using flow cytometry and observed around 15% cell death when Zn was added in both apical and basolateral media suggesting that enhanced zinc uptake may compromise cell viability (Figure 1D).

### Dengue Virus and Rotavirus Differentially Affect Permeability Barrier Functions

**Figure 2:**
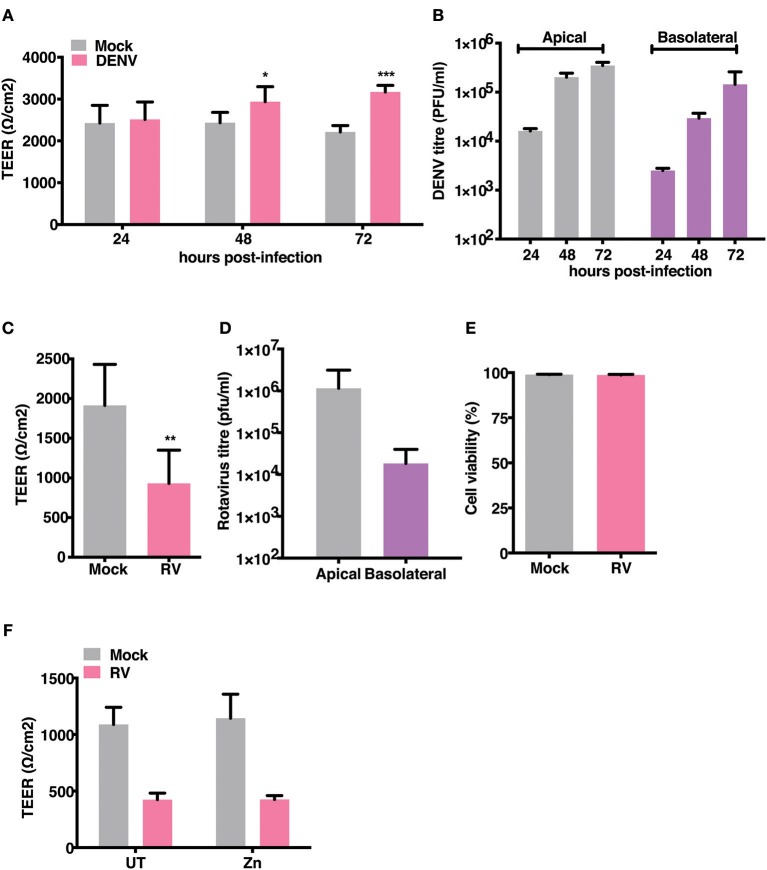
Effect of RV and DENV infection on epithelial barrier functions. Caco-2 cells were grown on transwell inserts for 4 days. Cells were infected with RV and DENV-2 at 0.5 and 10 MOI, respectively and TEER was measured. **(A)** TEER of DENV infected Caco-2 cells compared to mock measured till 72 h pi **(B)** Representative DENV titres determined by plaque assay and represented as pfu/ml at indicated time points. **(C)** TEER of RV infected Caco-2 cells compared to mock at 16 h pi **(D)** Representative RV titres determined by plaque assay and represented as pfu/ml at 16 h pi. **(E)** Viability of Caco-2 cells infected with RV as compared to mock measured by live dead staining using flow cytometry **(F)** TEER at 16 h pi of Caco-2 cells grown in transwells and infected with RV followed by addition of 50 μM ZnSO_4_. Data are from at least two independent experiments and represent mean ± SD. *p < 0.05, **p < 0.01, ***p < 0.001.

Gastrointestinal bleeding is a hallmark of severe dengue disease and previous reports implicate inflammatory cytokines such as TNF-α as a major player in this event (31). However, primate models of dengue infection have shown gastrointestinal tract to be one of the sites of virus replication but there have been no investigations to assess the direct effect of dengue infection on gut barrier functions (14). We examined the effect of dengue infection on barrier integrity in Caco-2 cells grown on trans-wells. Cells were infected with DENV-2 serotype at 10 MOI and cultured for 3 days with TEER readings recorded every 24 hrs and supernatants collected from apical and basolateral chambers for estimating viral titers. We observed no effect on TEER in cells infected with DENV up to 72 h pi. Surprisingly, dengue infected cells showed a significantly higher TEER values at later stages infection (Figure 2A). Despite lack of any negative effect on barrier functions, dengue virus was detected both in the apical and basolateral chamber of infected cells as early as 24 h pi and the virus titers steadily increased up to 72 h pi (Figure 2B). These results suggest that dengue virus does not directly disrupt epithelial barrier functions and is capable of exiting infected cells from the basolateral membranes. We used rotavirus (RV), which has been showed to infect Caco-2 cells and disrupt barrier functions by previous studies (32), as a positive control and observed that TEER was disrupted by RV infection at 16 h pi (Figures 2C,D) further confirming that these cells were not resistant to barrier disruption. We measured cell viability in rotavirus infection at 16 h pi by live-dead staining using flow cytometry and did not find any difference between mock and RV infection suggesting that factors other than cytotoxicity are responsible for disruption in the TEER (Figure 2E). Many Zn supplementation trials suggest that Zn therapy is beneficial in reducing the duration and severity in children infected with RV (33). Since Zn addition alone did not impact permeability barrier functions under our experimental conditions, we were interested in assessing the function of zinc on barrier functions in the context of RV infection. We next tested if addition of zinc into the medium 1 h after virus adsorption would prevent barrier disruption induced by RV. Cells were infected with 0.5 MOI of RV followed by apical treatment with 50 μM ZnSO_4_ till 16 h pi. TEER dropped by about 60% in RV-infected cells at 16 h pi but infected cells treated with Zn did not show any improvement in the TEER suggesting Zn did not rescue damaged barrier in cells post- infection in RV infected cells (Figure 2F).

### Zinc Chelation Specifically Inhibits Flavivirus Infection

**Figure 3:**
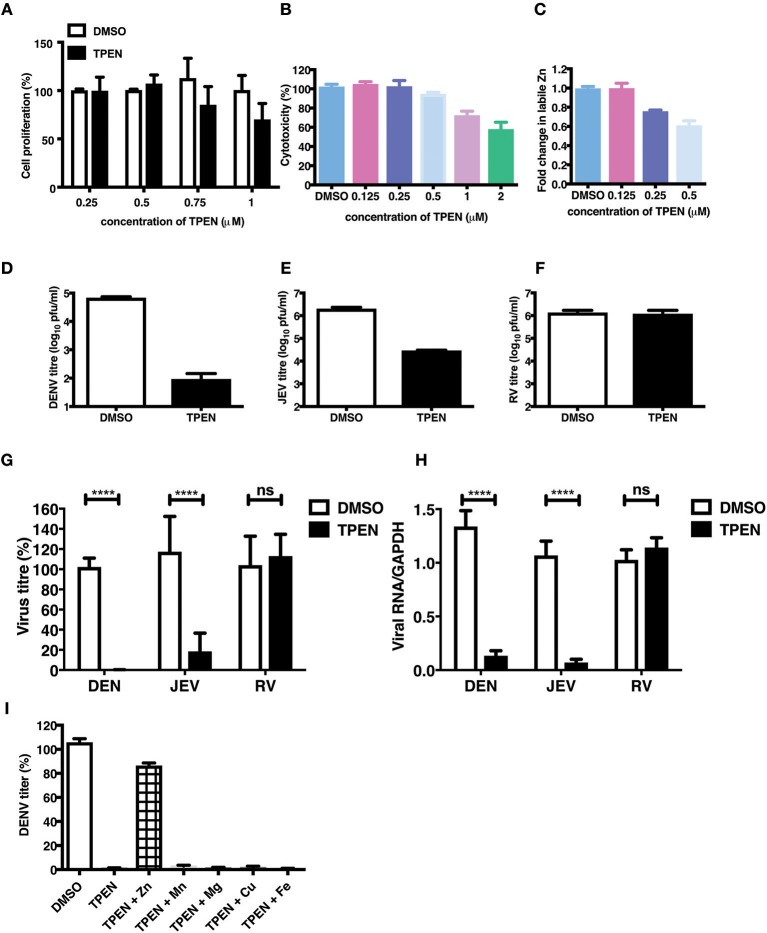
Zinc depletion specifically inhibits flavivirus infection. Caco-2 cells were treated with indicated concentrations of TPEN and incubated for 24 h. Cell proliferation **(A)** and cytotoxicity **(B)** was measured as described in the methods section. **(C)** Caco-2 cells were treated with indicated concentrations of TPEN and incubated for 4 h and amount of zinc depletion was measured by FACS using ZP-1. **(D)** Caco-2 cells were infected with DENV (10 MOI) or **(E)** JEV (3 MOI) or **(F)** RV (0.5 MOI) followed by treatment with TPEN at 0.5 μM. Viral titers in the culture supernatants were determined by plaque assay at 24 h pi for DENV and JEV; at 16 h pi for RV. Representative experiments are shown in D-F. Compilation of data from two or more experiments is shown for viral titers **(G)** and RT-PCR **(H)**. **(I)** Caco-2 cells infected with DENV were treated with TPEN after virus adsorption for 4 h followed by addition of 10 μM of ZnSO_4_/MnCl_2_/MgCl_2_/CuCl_2_/FeSO_4_ into the media. Viral titres were determined at 24 h post-addition of salts. All the data are from at least two independent experiments and presented as mean ± SD. ****p < 0.0001. ns, not significant.

The essential role of Zn in cellular functions has been demonstrated by many studies by using TPEN, a cell-permeable Zn chelator (34–36). We sought to mimic zinc deficiency *in vitro* using TPEN and determine the effect of Zn depletion on DENV infection. Many previous studies have shown around 70–80% depletion in labile Zn levels using high concentrations of TPEN for short duration (37, 38). We first measured the effect of increasing concentrations of TPEN on cell proliferation. Cells were treated with 0.25, 0.5, 0.75, and 1 μM TPEN for 24 h and cell proliferation was assessed. We observed that TPEN concentration above 0.5 μM affected cell proliferation (Figure 3A). Similarly, TPEN treatment above 0.5 μM showed cytotoxic effect and hence all further experiments were performed with 0.5 μM TPEN (Figure 3B). We estimated zinc depletion by FACS by staining with zinc fluorophore, ZP-1, after treatment with 0.125, 0.25, and 0.5 μM of TPEN for 4 h and found around 20 and 40% reduction in the mean fluorescence intensity of ZP-1 with 0.25 and 0.5 μM TPEN, respectively (Figure 3C). Next, Caco-2 cells were infected with DENV (10 MOI) or JEV (3 MOI) or RV (0.5 MOI) and after 1 h of virus adsorption, cells were cultured in serum-free medium containing DMSO (vehicle control) or 0.5 μM TPEN. Viral titers in the supernatant were measured by plaque assay at 24 h pi (for DENV and JEV) or 16 h pi (for RV). Zinc depletion by TPEN led to a substantial reduction in viral titers for both flaviviruses, DENV and JEV, but had no effect on RV titers in Caco-2 cells (Figures 3D–H). These results indicate that zinc chelation by TPEN, which results in moderate depletion in the labile zinc levels under our experimental conditions, has a drastic negative effect on flaviviruses but not on rotavirus suggesting that the cellular zinc levels have a differential effect on the life-cycle of positive (DENV and JEV) and double-stranded (RV) RNA viruses. TPEN binding has been shown to be highly specific to zinc; however, it has also been reported to bind to other metal ions albeit with far lower affinities (39). To further verify the specific role of zinc chelation by TPEN in DENV replication, we performed supplementation experiments after TPEN treatment. We assessed whether the effect of TPEN can be blocked by addition of salts of Zn or other divalent cations such as magnesium (Mg), manganese (Mn), copper (Cu), or iron (Fe). Caco-2 cells were treated with TPEN for 4 h after infection with DENV-2. After 4 h treatment, the culture medium was supplemented with 10 μM of ZnSO_4_/MnCl_2_/MgCl_2_/CuCl_2_/FeSO_4_ and viral titers in the supernatant was measured by plaque assay at 24 h pi. We observed that only ZnSO_4_ blocked the inhibitory effect of zinc chelation (Figure 3I). These results confirm that the inhibitory effect of TPEN is specifically due to zinc chelation.

### Zinc Chelation Affects DENV RNA Replication

**Figure 4:**
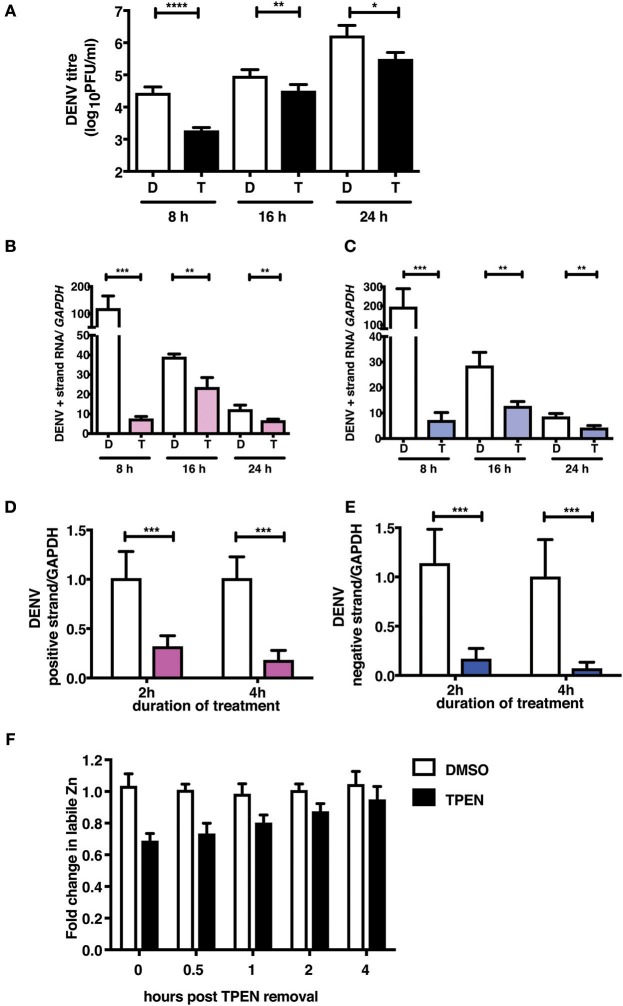
Zinc chelation perturbs DENV replication. **(A)** Caco-2 cells were infected with DENV and TPEN was added at 8, 16 and 24 h pi. Viral titers in the culture supernatants were determined after 16 h of treatment. **(B)** DENV positive and **(C)** negative strand levels were quantitated by qRT-PCR. Data are presented as fold increase is relative to the samples collected at the time-of-addition of TPEN. Caco-2 cells were treated with DMSO or TPEN (0.5 μM) for 2 and 4 h pi. After treatment media was replaced with serum-free medium and cells were collected at 24 h pi for estimation of **(D)** positive and **(E)** negative strand RNA by qRT-PCR. Data are presented as relative fold change in positive or negative strand RNA between DMSO and TPEN treatment. GAPDH mRNA levels were used for normalization. **(F)** Caco-2 cells were treated with DMSO or TPEN (0.5 μM) for 4 h pi. After treatment, media was replaced with serum-free medium and recovery in labile zinc levels were estimated by FACS at indicated time points after media addition by ZP-1 staining. Data are from at least two independent experiments performed with two or more replicates. Data are presented as mean ± SD. *p < 0.05, **p < 0.01, ***p < 0.001, ****p < 0.0001.

To further demonstrate the mechanism of action of TPEN treatment in DENV infection, we performed time-of-addition experiments. We added TPEN at 8, 16, and 24 h pi and viral titers were measured after 16 h treatment at each time point. We observed a significant reduction in viral titers when TPEN was added at any of the time-points indicated above, however, the reduction in viral titers was more drastic at early stages of viral infection (Figure 4A). DENV is a positive strand RNA virus which replicates via negative strand RNA intermediates. Therefore, estimating the quantity of negative strand RNA is a direct measure of virus replication. We next measured the positive and negative strand viral RNA in total RNA isolated from cells from the time-of-addition experiments by RT-PCR. The amount of positive and negative strand RNA increased to over hundred-fold in DMSO treated samples after 16 h of treatment relative to the time of treatment initiation, however, there was very minimal increase in viral RNA in TPEN-treated samples suggesting that zinc chelation affected viral replication (Figures 4B,C).

We further confirmed the effect of zinc chelation at early stages of viral replication by treating cells for short duration (2 and 4 h) after virus adsorption. Cells were further cultured in the absence of the inhibitor for 24 h pi. Total RNA was isolated and positive and negative strand RNA was measured. We observed a significant decrease in the amount of plus and minus strand RNA in TPEN treated cells in a time-dependent fashion where >80% reduction in RNA was observed in cells treated with TPEN for 4 h (Figures 4D,E). This data clearly demonstrates the requirement of cellular zinc or zinc-dependent processes for DENV RNA replication post-entry. We further estimated the time required for recovery of labile zinc pools after removal of TPEN in the medium. We observed that the labile zinc levels recovered to about 90% of DMSO controls within 2 h of replacing the medium suggesting that transient zinc chelation may activate pathways that counter dengue replication (Figure 4F).

### TPEN Pre-treatment Blocks DENV Infection

**Figure 5:**
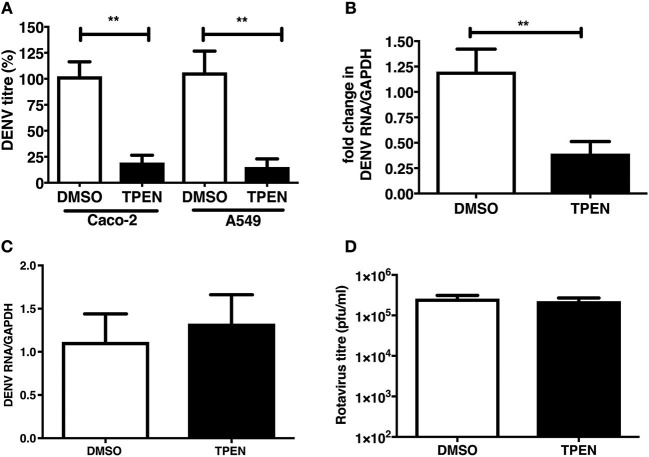
Zinc chelation prior to DENV infection inhibits DENV infection. **(A)** Caco-2 or A549 cells were treated with DMSO or TPEN (0.5 μM) for 4 h prior to DENV infection. Infected cells were cultured for 24 h in the absence of TPEN and viral titers in the supernatant was measured by plaque assay and **(B)** DENV RNA was quantitated by qRT-PCR. **(C)** Cells pre-treated with TPEN and infected with DENV were collected by trypsinization 1 h pi and total RNA was isolated to measure internalized viral RNA by qRT-PCR. **(D)** Caco-2 cells were treated with DMSO or TPEN (0.5 μM) for 4 h prior to RV infection. Infected cells were cultured for 16 h in the absence of TPEN and viral titers in the supernatant was measured by plaque assay. All the data are from at least two independent experiments with triplicate samples. Data are presented as mean ± SD. **p < 0.01.

We were next interested in determining whether zinc chelation prior to dengue infection has any effect on virus infection. Caco-2 and A549 cells were pre-treated with TPEN for 4 h prior to DENV infection. Cells were infected as above and cultured for 24 h in the absence of TPEN. Virus titers in the supernatant was measured by plaque assays at 24 h pi. We observed a significant reduction in DENV titers in both the cell lines and DENV RNA levels were also reduced upon TPEN pre-treatment in Caco-2 cells suggesting that perturbing zinc homeostasis prior to infection also affects DENV infection (Figures 5A,B). This effect was not due to defect in cellular entry of the virus as the amount of internalized viral RNA after 1 h of virus adsorption was not different between mock-treated and zinc-depleted cells (Figure 5C). Pre-treating cells with TPEN had no effect on rotavirus infection (Figure 5D). These data suggest that zinc chelation specifically affects dengue virus infection.

### Zinc Chelation Activates Stress Response Pathways

**Figure 6:**
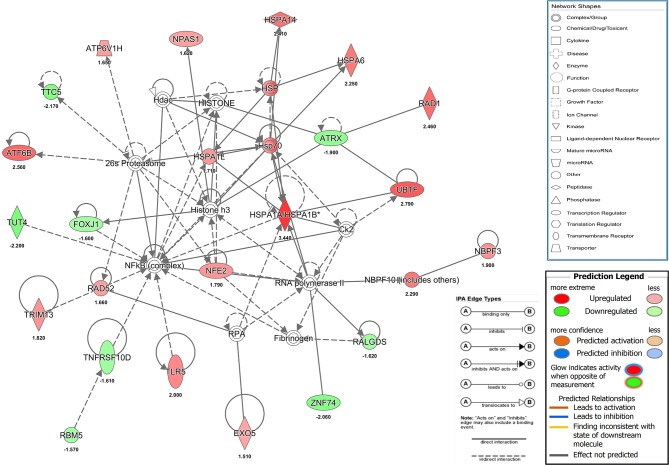
Network analysis from RNA seq. Figure shows one of the functional networks (post-translational modification and protein folding) identified from the list of differentially expressed genes obtained from DMSO and TPEN treatment conditions by RNA seq analysis using ingenuity pathway analysis tool. Please see materials and methods section for further details.

Our results showed that a transient and reversible zinc chelation was sufficient to perturb DENV replication suggesting that chelating free zinc may modulate cellular pathways that negatively affect DENV replication. To gain further insights into the molecular mechanism behind zinc depletion-induced inhibition of DENV replication, we treated cells with TPEN for 4 h and processed total RNA for RNA seq analysis. One eighty three genes (99 genes were downregulated and 84 genes were upregulated) showed differential expression upon treatment (Supplementary Table 2 and Supplementary Figure 2). The differential gene expression data obtained from RNA-seq was further analyzed by Ingenuity Pathway Analysis (IPA) for identifying interaction networks, molecular and cellular functions and pathways that are modulated upon zinc chelation. As expected from the diverse physiological functions regulated by zinc, the list of genes whose expression was altered due to zinc chelation was clustered around physiological functions such as post-translational modification, protein folding and signal transduction pathways (Supplementary Table 3). Network analysis of differentially expressed genes by IPA analysis further confirmed enrichment of genes involved in post-translational modification and protein folding which has 35 nodes includes 24 focus genes (Figure 6). Interestingly, induction of heat shock proteins and NF-κB activation emerged as one of the major nodes in the network analysis (Figure 6) suggesting that zinc chelation may lead to activation of ER stress and NF-κB pathway in addition of stress response involving heat shock proteins.

### Zinc Chelation Induces Antiviral Response Due to ER Stress and NF-κB Activation

**Figure 7:**
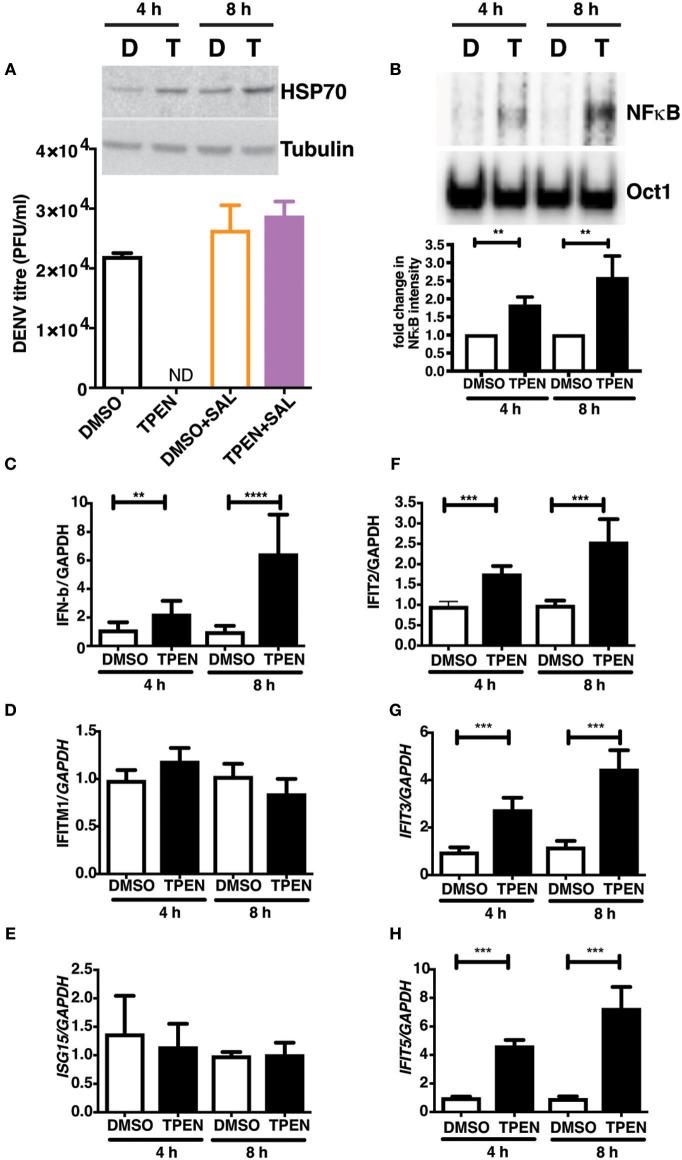
Zinc chelation induces antiviral response. **(A)** Inset: Caco-2 cells were treated with 0.5 μM TPEN and DMSO for 4 and 8 h and used for western blot analysis of HSP70. Caco-2 cells were treated with DMSO or TPEN (0.5 μM) in the presence of 50 μM Salubrinal after infection and virus titers were determined at 24 h pi. **(B)** Caco-2 cells were treated with DMSO or TPEN (0.5 μM) for 4 or 8 h. Nuclear extracts were prepared and NF-κB DNA binding activity was assessed by EMSA. Oct1 served as a control for normalization. **(C–G)** Caco-2 cells were treated with DMSO or TPEN (0.5 μM) for 4 or 8 h. Total RNA was isolated and transcript levels of indicated genes were quantitated by RT-PCR. All the data are from at least two independent experiments with triplicate samples. Data are presented as mean ± SD. **p < 0.01, ***p < 0.001, ****p < 0.0001.

RNA seq analysis suggested that zinc chelation may activate ER stress response and NF-κB and its downstream targets which may block dengue virus replication. We next tested if blocking unfolded protein response would rescue the inhibitory effect of zinc chelation. Cells were infected with DENV and cultured in medium containing DMSO or TPEN in the presence of Salubrinal, an inhibitor of unfolded protein response (UPR) (40). Blocking UPR by Salubrinal rescued from the inhibition observed with zinc chelation suggesting a connection between zinc homeostasis, UPR and antiviral response (Figure 7A). To further confirm the induction of antiviral response due to zinc chelation, cells were treated with TPEN for 4 and 8 h and nuclear extracts were prepared to measure NF-κB activity by electrophoretic mobility shift assays (EMSA). We observed a time-dependent increase in NF-κB activity in TPEN-treated cells (Figure 7A). To further confirm the functional relevance of NF-κB activation, cells were treated with TPEN for 4 and 8 h and relative transcript levels of interferon-β and some of its downstream effectors were measured by RT-PCR. Zinc chelation by TPEN led to induction in IFN-β mRNA as early as 4 h post-treatment which further increased at 8 h post-treatment (Figure 7B). We further measured the mRNA levels of interferon-responsive genes namely, interferon-induced transmembrane protein 1 (IFITM1), interferon-stimulated gene 15 (ISG15), interferon-induced protein with tetratricopeptide repeats 2, 3, and 5 (IFIT2, IFIT3, and IFIT5) (Figures 7C–H). Of all these genes, only IFIT2, IFIT3, and IFIT5 showed induction, however, IFIT5 showed maximum induction as compared to IFIT2 and IFIT3 (Figures 7F–H). Interestingly, IFIT5 was one of the genes upregulated upon TPEN treatment in the RNA seq data (Supplementary Table 2). Our results suggest that zinc chelation activates NF-κB and generates an antiviral state by inducing a specific subset of interferon-mediated antiviral signaling that renders cells resistant to DENV infection.

## Discussion

In this study, we focused on the effect of rotavirus and dengue virus infection on epithelial junctions and the importance of zinc in maintaining the barrier functions in Caco-2 cells in the context of virus infection. Zinc supplementation trials have shown that oral zinc as a supplement in diarrhea is beneficial in children over 6 months of age in regions where zinc deficiency is prevalent (33) indicating that chronic zinc deficiency could be corrected by zinc supplementation. However, whether zinc would be beneficial in enteric infections under normal conditions has been inconclusive. Furthermore, the effect of zinc supplementation in rotavirus diarrhea has been inconsistent (41–43). In our *in vitro* study, we show that zinc supplementation post-infection had no effect on viral titres or barrier disruption in rotavirus-infected cells suggesting that the effect of zinc *in vivo* may involve its effect on immune response or other antiviral functions. Gastrointestinal manifestations are commonly observed in severe cases of dengue (44, 45) and the immunopathological nature of severe dengue disease suggests that these manifestations are a result of cytokine storm. However, replication of dengue virus in the midgut epithelial cells of mosquitos and isolation of viable virus from the intestine of experimentally-infected rhesus monkeys suggest that gut epithelial cells may be one of the active sites of DENV replication and virus dissemination (13, 14). We show that Caco-2 cells were not only permissive to dengue infection, but the virus was released from both the apical and basolateral surfaces without disruption of cellular junctions. Based on these observations we propose a crucial role for epithelial cells in dengue pathogenesis which needs to be further verified in relevant and immunocompetent animal models.

Zinc salts have been shown to inhibit some of the RNA viruses using *in vitro* assays or infection systems (4–9). Zinc has been shown to alter various stages of viral infection as in the case of poliovirus and other picornaviruses and hepatitis C virus where zinc salts were shown to inhibit viral protease activity (46, 47). Contrary to many of the aforesaid reports, zinc chelation was shown to inhibit the alfalfa mosaic virus RNA-dependent-RNA polymerase (RdRp) activity (48). The reverse transcriptase of RNA tumor viruses is a zinc metalloenzyme and zinc chelation was shown to inhibit the enzyme (49, 50). Similarly, zinc chelation also affected the RdRp activity of influenza viruses and the 2A proteinase of human rhinovirus (51, 52). Therefore, the role of zinc appears to be as diverse as the strategies of replication adopted by the RNA viruses from different families. We took the approach of mimicking moderate zinc deficiency conditions by zinc chelation using TPEN to identify the effect of zinc-dependent viral and cellular functions on RNA virus infections. We observed that zinc depletion by TPEN inhibited DENV and JEV infection but not RV infection. Time-of-addition experiments indicated that zinc plays an essential role at early stages of viral RNA replication, post-entry and post-uncoating, as observed by a drastic reduction in the synthesis of negative and positive strand RNA in TPEN-treated cells. Additionally, zinc chelation for only first 4 h of infection led to near-complete inhibition of virus replication. The inhibitory effect of TPEN was specific to Zn chelation because only Zn supplementation could reverse the effect and other divalent cations failed to do so.

Our RNA seq analysis provided insights into the molecular functions perturbed due to transient and moderate zinc depletion. Other than post-translational modification and protein folding, many zinc-finger proteins, which function as transcription factors, were dysregulated suggesting a global effect on host transcription and translation. Therefore, it is not surprising that addition of TPEN even at 24 h post-infection was able to significantly inhibit virus titers. However, these changes specifically affected flavivirus infection and had no effect on rotavirus suggesting that zinc homeostasis may regulate few viruses depending on the requirement of cellular zinc for completion of virus life-cycle. We further discovered that transient zinc chelation led to induction of an antiviral state in cells via induction of heat shock proteins and activation of NF-κB and upregulation of downstream effectors which inhibit DENV replication. Interferon-stimulated genes (ISGs) are a large group of genes which have diverse effects on viral infections and mostly act at early stages of virus life-cycle (53). The IFIT proteins are a family of antiviral proteins that form complexes to target viral RNA and inhibit translation (54–56). Although DENV has been shown to evade interferon responses, induction of ISGs at very early stages before the accumulation of viral proteins that target some of the innate immune components may tip the balance in favor of innate immune responses and block viral RNA replication. Surprisingly, labile zinc pool was restored within 2 h of removing the zinc chelator suggesting that transient zinc depletion activates the stress response involving heat shock proteins from the Hsp70 family and an antiviral state due to NF-κB activation. Interestingly, Hsp70 family members play a necessary role in dengue replication and Hsp70 has been proposed as an antiviral target (57, 58). The molecular mechanism linking zinc depletion to Hsp70 remains to be elucidated.

Zinc is an acute-phase reactant and zinc levels are redistributed during infections. It is plausible that this redistribution may create a transient state of zinc deficiency during acute viral infections which may indirectly protect cells by inducing an antiviral state. It has been suggested that pathogens may actively alter zinc levels and zinc chelation with TPEN was shown to improve survival of mice infected with *Aspergillus fumigatus* (59). In the case of HIV, zinc chelation by TPEN was shown to inhibit APOBEC3 degradation by HIV-1Vif and made the virus susceptible to the antiviral activity of APOBEC3 (60). These studies suggest that zinc chelation can be an attractive antimicrobial option. Considering that zinc ion was co-crystallized with dengue polymerase, the non-structural protein 5 (NS5) (11), we speculate that the DENV NS5 may utilize cellular zinc for viral RNA replication. It would be interesting to further investigate whether zinc homeostasis is altered during DENV replication. Therefore, cellular or tissue zinc homeostasis may also determine the efficiency with which pathogens replicate and disseminate *in vivo*. We speculate that in the case of acute viral infections, strategies to transiently block zinc redistribution during viremic stages may inhibit viruses that depend on cellular zinc pools for replication. This would provide a window for the immune system to gain an upper hand and control viral infection.

## Data Availability Statement

The datasets generated for this study has been deposited in the Gene expression Omnibus (GEO) database with accession number GSE135873.

## Author Contributions

MK, NK, AP, SSB, and RG performed experiments and analyzed data. SS and SB designed experiments and analyzed the data. GM conceived the study, designed experiments, performed experiments, analyzed data, and wrote the manuscript. All the authors have reviewed and approved the final version of the manuscript.

### Conflict of Interest

The authors declare that the research was conducted in the absence of any commercial or financial relationships that could be construed as a potential conflict of interest.

## References

[1] Liu CH Zhou L Chen G Krug RM. Battle between influenza A virus and a newly identified antiviral activity of the PARP-containing ZAPL protein. Proc Natl Acad Sci USA. (2015) 112:14048–53. 10.1073/pnas.1509745112 26504237 PMC4653199

[2] Chen SC Jeng KS Lai MMC. Zinc finger-containing cellular transcription corepressor ZBTB25 promotes influenza virus RNA transcription and is a target for zinc ejector drugs. J Virol. (2017) 91:e00842–17. 10.1128/JVI.00842-17 28768860 PMC5625503

[3] Read SA Parnell G Booth D Douglas MW George J Ahlenstiel G. The antiviral role of zinc and metallothioneins in hepatitis C infection. J Viral Hepat. (2018) 25:491–501. 10.1111/jvh.12845 29239069

[4] Gaudernak E Seipelt J Triendl A Grassauer A Kuechler E. Antiviral effects of pyrrolidine dithiocarbamate on human rhinoviruses. J Virol. (2002) 76:6004–15. 10.1128/JVI.76.12.6004-6015.2002 12021333 PMC136215

[5] Merluzzi VJ Cipriano D McNeil D Fuchs V Supeau C Rosenthal AS. Evaluation of zinc complexes on the replication of rhinovirus 2 *in vitro*. Res Commun Chem Pathol Pharmacol. (1989) 66:425–40. 2558406

[6] Si X McManus BM Zhang J Yuan J Cheung C Esfandiarei M. Pyrrolidine dithiocarbamate reduces coxsackievirus B3 replication through inhibition of the ubiquitin-proteasome pathway. J Virol. (2005) 79:8014–23. 10.1128/JVI.79.13.8014-8023.2005 15956547 PMC1143712

[7] te Velthuis AJ van den Worm SH Sims AC Baric RS Snijder EJ van Hemert MJ. Zn(2+) inhibits coronavirus and arterivirus RNA polymerase activity *in vitro* and zinc ionophores block the replication of these viruses in cell culture. PLoS Pathog. (2010) 6:e1001176. 10.1371/journal.ppat.1001176 21079686 PMC2973827

[8] Yamasaki S Sakata-Sogawa K Hasegawa A Suzuki T Kabu K Sato E. Zinc is a novel intracellular second messenger. J Cell Biol. (2007) 177:637–45. 10.1083/jcb.200702081 17502426 PMC2064209

[9] Kaushik N Subramani C Anang S Muthumohan R Shalimar Nayak B. Zinc salts block hepatitis E virus replication by inhibiting the activity of viral RNA-dependent RNA polymerase. J Virol. (2017) 91:e00754–17. 10.1128/JVI.00754-17 28814517 PMC5640865

[10] Stanaway JD Shepard DS Undurraga EA Halasa YA Coffeng LE Brady OJ. The global burden of dengue: an analysis from the Global Burden of Disease Study 2013. Lancet Infect Dis. (2016) 16:712–23. 10.1016/S1473-3099(16)00026-8 26874619 PMC5012511

[11] Yap TL Xu T Chen YL Malet H Egloff MP Canard B. Crystal structure of the dengue virus RNA-dependent RNA polymerase catalytic domain at 1.85-angstrom resolution. J Virol. (2007) 81:4753–65. 10.1128/JVI.02283-06 17301146 PMC1900186

[12] El Sahili A Lescar J. Dengue virus non-structural protein 5. Viruses. (2017) 9:E91. 10.3390/v9040091 28441781 PMC5408697

[13] Salazar MI Richardson JH Sanchez-Vargas I Olson KE Beaty BJ. Dengue virus type 2: replication and tropisms in orally infected Aedes aegypti mosquitoes. BMC Microbiol. (2007) 7:9. 10.1186/1471-2180-7-9 17263893 PMC1797809

[14] Marchette NJ Halstead SB Nash DR Stenhouse AC. Recovery of dengue viruses from tissues of experimentally infected rhesus monkeys. Appl Microbiol. (1972) 24:328–33. 4627963 10.1128/am.24.3.328-333.1972 PMC376519

[15] Agrawal T Schu P Medigeshi GR. Adaptor protein complexes-1 and 3 are involved at distinct stages of flavivirus life-cycle. Sci Rep. (2013) 3:1813. 10.1038/srep01813 23657274 PMC3648799

[16] Agrawal T Sharvani V Nair D Medigeshi GR. Japanese encephalitis virus disrupts cell-cell junctions and affects the epithelial permeability barrier functions. PLoS ONE. (2013) 8:e69465. 10.1371/journal.pone.0069465 23894488 PMC3722119

[17] Medigeshi GR Kumar R Dhamija E Agrawal T Kar M. N-desmethylclozapine, fluoxetine, and salmeterol inhibit postentry stages of the dengue virus life cycle. Antimicrob Agents Chemother. (2016) 60:6709–18. 10.1128/AAC.01367-16 27572397 PMC5075077

[18] Kar M Singla M Chandele A Kabra SK Lodha R Medigeshi GR Dengue virus entry and replication does not lead to productive infection in platelets. Open Forum Infect Dis. (2017) 4:ofx051 10.1093/ofid/ofx051 28491890 PMC5420081

[19] Gurukumar KR Priyadarshini D Patil JA Bhagat A Singh A Shah PS. Development of real time PCR for detection and quantitation of Dengue viruses. Virol J. (2009) 6:10. 10.1186/1743-422X-6-10 19166574 PMC2651855

[20] Jothikumar N Kang G Hill VR Broadly reactive TaqMan® assay for real-time RT-PCR detection of rotavirus in clinical and environmental samples. J Virol Methods. (2009) 155:126–31. 10.1016/j.jviromet.2008.09.025 18951923

[21] Mukherjee T Chatterjee B Dhar A Bais SS Chawla M Roy P. A TNF-p100 pathway subverts noncanonical NF-kappaB signaling in inflamed secondary lymphoid organs. EMBO J. (2017) 36:3501–16. 10.15252/embj.201796919 29061763 PMC5709727

[22] Patel RK Jain M. NGS QC Toolkit: a toolkit for quality control of next generation sequencing data. PLoS ONE. (2012) 7:e30619. 10.1371/journal.pone.0030619 22312429 PMC3270013

[23] Trapnell C Pachter L Salzberg SL. TopHat: discovering splice junctions with RNA-Seq. Bioinformatics. (2009) 25:1105–11. 10.1093/bioinformatics/btp120 19289445 PMC2672628

[24] Trapnell C Roberts A Goff L Pertea G Kim D Kelley DR. Differential gene and transcript expression analysis of RNA-seq experiments with TopHat and Cufflinks. Nat Protoc. (2012) 7:562–78. 10.1038/nprot.2012.016 22383036 PMC3334321

[25] de Hoon MJ Imoto S Nolan J Miyano S. Open source clustering software. Bioinformatics. (2004) 20:1453–4. 10.1093/bioinformatics/bth078 14871861

[26] Saldanha AJ. Java treeview–extensible visualization of microarray data. Bioinformatics. (2004) 20:3246–8. 10.1093/bioinformatics/bth349 15180930

[27] Wang Y Mumm JB Herbst R Kolbeck R Wang Y. IL-22 increases permeability of intestinal epithelial tight junctions by enhancing claudin-2 expression. J Immunol. (2017) 199:3316–25. 10.4049/jimmunol.1700152 28939759

[28] Ren H Birch NP Suresh V. An optimised human cell culture model for alveolar epithelial transport. PLoS ONE. (2016) 11:e0165225. 10.1371/journal.pone.0165225 27780255 PMC5079558

[29] Heijink IH Brandenburg SM Noordhoek JA Postma DS Slebos DJ van Oosterhout AJ. Characterisation of cell adhesion in airway epithelial cell types using electric cell-substrate impedance sensing. Eur Respir J. (2010) 35:894–903. 10.1183/09031936.00065809 19741028

[30] Verhoeckx K Cotter P López-Expósito I Kleiveland C Lea T Mackie A editors. The Impact of Food Bioactives on Health: in vitro and ex vivo Models. Cham: Springer (2015). 10.1007/978-3-319-16104-4 29787039

[31] Watanabe S Chan KW Wang J Rivino L Lok SM Vasudevan SG. Dengue virus infection with highly neutralizing levels of cross-reactive antibodies causes acute lethal small intestinal pathology without a high level of viremia in mice. J Virol. (2015) 89:5847–61. 10.1128/JVI.00216-15 25787279 PMC4442448

[32] Dickman KG Hempson SJ Anderson J Lippe S Zhao L Burakoff R. Rotavirus alters paracellular permeability and energy metabolism in Caco-2 cells. Am J Physiol Gastrointest Liver Physiol. (2000) 279:G757–66. 10.1152/ajpgi.2000.279.4.G757 11005763

[33] Lazzerini M Wanzira H. Oral zinc for treating diarrhoea in children. Cochrane Database Syst Rev. (2016) 12:Cd005436. 10.1002/14651858.CD005436.pub5 27996088 PMC5450879

[34] Homma K Fujisawa T Tsuburaya N Yamaguchi N Kadowaki H Takeda K. SOD1 as a molecular switch for initiating the homeostatic ER stress response under zinc deficiency. Mol Cell. (2013) 52:75–86. 10.1016/j.molcel.2013.08.038 24076220

[35] Kawamata T Horie T Matsunami M Sasaki M Ohsumi Y. Zinc starvation induces autophagy in yeast. J Biol Chem. (2017) 292:8520–30. 10.1074/jbc.M116.762948 28264932 PMC5437255

[36] Shumaker DK Vann LR Goldberg MW Allen TD Wilson KL. TPEN, a Zn2+/Fe2+ chelator with low affinity for Ca2+, inhibits lamin assembly, destabilizes nuclear architecture and may independently protect nuclei from apoptosis *in vitro*. Cell Calcium. (1998) 23:151–64. 10.1016/S0143-4160(98)90114-2 9601611

[37] Rahal ON Fatfat M Hankache C Osman B Khalife H Machaca K. Chk1 and DNA-PK mediate TPEN-induced DNA damage in a ROS dependent manner in human colon cancer cells. Cancer Biol Ther. (2016) 17:1139–48. 10.1080/15384047.2016.1235658 27690730 PMC5137490

[38] Zhu B Wang J Zhou F Liu Y Lai Y Wang J. Zinc depletion by TPEN induces apoptosis in human acute promyelocytic nb4 cells. Cell Physiol Biochem. (2017) 42:1822–36. 10.1159/000479539 28750402

[39] Hyun HJ Sohn JH Ha DW Ahn YH Koh JY Yoon YH. Depletion of intracellular zinc and copper with TPEN results in apoptosis of cultured human retinal pigment epithelial cells. Invest Ophthalmol Visual Sci. (2001) 42:460–5. 11157883

[40] Boyce M Bryant KF Jousse C Long K Harding HP Scheuner D. A selective inhibitor of eIF2alpha dephosphorylation protects cells from ER stress. Science. (2005) 307:935–9. 10.1126/science.1101902 15705855

[41] Bhatnagar S Bahl R Sharma PK Kumar GT Saxena SK Bhan MK. Zinc with oral rehydration therapy reduces stool output and duration of diarrhea in hospitalized children: a randomized controlled trial. J Pediatr Gastroenterol Nutr. (2004) 38:34–40. 10.1097/00005176-200401000-00010 14676592

[42] Dalgic N Sancar M Bayraktar B Pullu M Hasim O. Probiotic, zinc and lactose-free formula in children with rotavirus diarrhea: are they effective? Pediatr Int. (2011) 53:677–82. 10.1111/j.1442-200X.2011.03325.x 21261786

[43] Jiang CX Xu CD Yang CQ. [Therapeutic effects of zinc supplement as adjunctive therapy in infants and young children with rotavirus enteritis]. Zhongguo Dang Dai Er Ke Za Zhi. (2016) 18:826–30. 10.7499/j.issn.1008-8830.2016.09.008 27655538 PMC7389982

[44] Ooi ET Ganesananthan S Anil R Kwok FY Sinniah M. Gastrointestinal manifestations of dengue infection in adults. Med J Malaysia. (2008) 63:401–5. 19803300

[45] Vejchapipat P Theamboonlers A Chongsrisawat V Poovorawan Y. An evidence of intestinal mucosal injury in dengue infection. South Asian J Trop Med Public Health. (2006) 37:79–82. 16771216

[46] Nicklin MJH Toyoda H Murray MG Wimmer E Proteolytic processing in the replication of polio and related viruses. Bio/Technology. (1986) 4:33–42. 10.1038/nbt0186-33

[47] Tedbury PR Harris M. Characterisation of the role of zinc in the hepatitis C virus NS2/3 auto-cleavage and NS3 protease activities. J Mol Biol. (2007) 366:1652–60. 10.1016/j.jmb.2006.12.062 17239391

[48] Quadt R Jaspars EM. Effect of removal of zinc on alfalfa mosaic virus RNA-dependent RNA polymerase. FEBS Lett. (1991) 278:61–2. 10.1016/0014-5793(91)80083-F 1993475

[49] Poiesz BJ Seal G Loeb LA. Reverse transcriptase: correlation of zinc content with activity. Proc Natl Acad Sci USA. (1974) 71:4892–6. 10.1073/pnas.71.12.4892 4140513 PMC434005

[50] Auld DS Kawaguchi H Livingston DM Vallee BL. RNA-dependent DNA polymerase (reverse transcriptase) from avian myeloblastosis virus: a zinc metalloenzyme. Proc Natl Acad Sci USA. (1974) 71:2091–5. 10.1073/pnas.71.5.2091 4134617 PMC388392

[51] Oxford JS Perrin DD. Inhibition of the particle-associated RNA-dependent RNA polymerase activity of influenza viruses by chelating agents. J General Virol. (1974) 23:59–71. 10.1099/0022-1317-23-1-59 4833601

[52] Sommergruber W Casari G Fessl F Seipelt J Skern T. The 2A proteinase of human rhinovirus is a zinc containing enzyme. Virology. (1994) 204:815–8. 10.1006/viro.1994.1599 7941352

[53] Schoggins JW Wilson SJ Panis M Murphy MY Jones CT Bieniasz P. A diverse range of gene products are effectors of the type I interferon antiviral response. Nature. (2011) 472:481–5. 10.1038/nature09907 21478870 PMC3409588

[54] Fleith RC Mears HV Leong XY Sanford TJ Emmott E Graham SC. IFIT3 and IFIT2/3 promote IFIT1-mediated translation inhibition by enhancing binding to non-self RNA. Nucleic Acids Res. (2018) 46:5269–85. 10.1093/nar/gky191 29554348 PMC6007307

[55] Johnson B VanBlargan LA Xu W White JP Shan C Shi PY. Human IFIT3 modulates IFIT1 RNA binding specificity and protein stability. Immunity. (2018) 48:487–99.e5. 10.1016/j.immuni.2018.01.014 29525521 PMC6251713

[56] Mears HV Sweeney TR. Better together: the role of IFIT protein-protein interactions in the antiviral response. J Gen Virol. (2018) 99:1463–77. 10.1099/jgv.0.001149 30234477

[57] Taguwa S Maringer K Li X Bernal-Rubio D Rauch JN Gestwicki JE. Defining Hsp70 subnetworks in dengue virus replication reveals key vulnerability in flavivirus infection. Cell. (2015) 163:1108–23. 10.1016/j.cell.2015.10.046 26582131 PMC4869517

[58] Howe MK Speer BL Hughes PF Loiselle DR Vasudevan S Haystead TA. An inducible heat shock protein 70 small molecule inhibitor demonstrates anti-dengue virus activity, validating Hsp70 as a host antiviral target. Antiviral Res. (2016) 130:81–92. 10.1016/j.antiviral.2016.03.017 27058774 PMC4955699

[59] Laskaris P Atrouni A Calera JA d'Enfert C Munier-Lehmann H Cavaillon J-M Administration of zinc chelators improves survival of mice infected with named-content genus-species named-content- Aspergillus fumigatus both in Monotherapy and in Combination with Caspofungin. Antimicrob Agents Chemother. (2016) 60:5631–9. 10.1128/AAC.00324-16 27401578 PMC5038287

[60] Xiao Z Ehrlich E Luo K Xiong Y Yu XF. Zinc chelation inhibits HIV Vif activity and liberates antiviral function of the cytidine deaminase APOBEC3G. FASEB J. (2007) 21:217–22. 10.1096/fj.06-6773com 17135358

