# Supplementary material for: Zinc Chelation Specifically Inhibits Early Stages of Dengue Virus Replication by Activation of NF-κB and Induction of Antiviral Response in Epithelial Cells
Source: Front Immunol. 2019 Oct 1;10:2347. doi: 10.3389/fimmu.2019.02347 (PMC6779808; doi:10.3389/fimmu.2019.02347)
Supplement: Supplementary file 1 [file Data_Sheet_1.PDF]

## Supplementary Table T1: List of RT-PCR primers used in the study

| Gene | Forward primer (5’-3’) | Reverse primer (5’-3’) |
|---|---|---|
| *GAPDH* | CCACTCCTCCACCTTTGAC | ACCCTGTTGCTGTAGCCA |
| *IFN-$\beta$* | AAACTCATGAGCAGTCTGCA | AGGAGATCTTCAGTTTCGGAGG |
| *IFITM1* | ACTCCGTGAAGTCTAGGGACA | TGTCACAGAGCCGAATACCAG |
| *ISG15* | AGATCACCCAGAAGATCG | TGTTATTCCTCACCAGGATG |
| *IFIT2* | ATAGGGCTCAGTATCCCCCA | TTGCTTTTTGTACCCGTCGC |
| *IFIT3* | AGAGCAGTTTGTTGAAGAAG | TTACTTTTGCCTTGTAGCAG |
| *IFIT5* | GAGTGAAATTCGTAAGGACAC | CCTTTTAGGTGTTTCACATAGG |

## Supplementary Table T2: List of differentially expressed genes identified by RNA seq analysis.

| TCON_ID | GENE_MODEL_ID | GENE_SYMBOL | CHROMOSOME | START | END | FPKM_DMSO | FPKM_TPEN | FOLD_EXP_DMSO | FOLD_EXP_TPEN | log2(fold_change) | p_value | EXPRESSION_STATUS | REFSEQ_ID | Gene Name |
|---|---|---|---|---|---|---|---|---|---|---|---|---|---|---|
| TCONS_00034820 | XLOC_015142 | AGFG1 | chr2 | 227472131 | 227561222 | 1.40 | 0.46 | 0.49 | -1.13 | -1.62 | 0.03 | DOWN-REGULATED | NM_001135188 | ArfGAP with FG repeats 1 |
| TCONS_00000877 | XLOC_000399 | AKR1A1 | chr1 | 45550782 | 45570051 | 13.33 | 3.34 | 3.74 | 1.74 | -2.00 | 0.02 | DOWN-REGULATED | NM_001202414 | aldo-keto reductase family 1, member A1 (aldehyde reductase) |
| TCONS_00010547 | XLOC_004662 | AMBRA1 | chr11 | 46396411 | 46594069 | 1.01 | 0.27 | 0.02 | -1.88 | -1.90 | 0.02 | DOWN-REGULATED | NM_001300731 | autophagy/beclin-1 regulator 1 |
| TCONS_00063904 | XLOC_028170 | AMMECR1 | chrX | 110194185 | 110456334 | 1.02 | 3.09 | 0.04 | 1.63 | 1.59 | 0.04 | UP-REGULATED | NM_001025580 | Alport syndrome, mental retardation, midface hypoplasia and elliptocytosis chromosomal region gene 1 |
| TCONS_00013663 | XLOC_006058 | AMN1 | chr12 | 31671136 | 31729174 | 2.82 | 10.69 | 1.50 | 3.42 | 1.92 | 0.00 | UP-REGULATED | NM_001113402 | antagonist of mitotic exit network 1 homolog (S. cerevisiae) |
| TCONS_00013667 | XLOC_006058 | AMN1 | chr12 | 31671136 | 31729174 | 0.31 | 2.04 | -1.68 | 1.03 | 2.71 | 0.02 | UP-REGULATED | NR_103523 | antagonist of mitotic exit network 1 homolog (S. cerevisiae) |
| TCONS_00059220 | XLOC_026021 | ANGPT1 | chr8 | 107249481 | 107498026 | 0.31 | 1.23 | -1.71 | 0.30 | 2.01 | 0.02 | UP-REGULATED | NM_001199859 | angiopoietin 1 |
| TCONS_00044995 | XLOC_019724 | ANKRD37 | chr4 | 185396685 | 185425985 | 3.59 | 14.03 | 1.85 | 3.81 | 1.96 | 0.00 | UP-REGULATED | NM_181726 | ankyrin repeat domain 37 |
| TCONS_00025182 | XLOC_011391 | ANKRD40 | chr17 | 50693189 | 50707909 | 26.61 | 8.74 | 4.73 | 3.13 | -1.61 | 0.00 | DOWN-REGULATED | NM_052855 | ankyrin repeat domain 40 |
| TCONS_00036910 | XLOC_016073 | AP5S1 | chr20 | 3820523 | 3825307 | 1.02 | 3.65 | 0.03 | 1.87 | 1.84 | 0.01 | UP-REGULATED | NM_001204447 | adaptor related protein complex 5 sigma 1 subunit(AP5S1) |
| TCONS_00062634 | XLOC_027625 | ARMCX5 | chrX | 102599167 | 102717733 | 3.08 | 1.00 | 1.62 | 0.01 | -1.62 | 0.05 | DOWN-REGULATED | NM_001168478 | armadillo repeat containing, X-linked 5 |
| TCONS_00008553 | XLOC_003762 | ARNTL | chr11 | 13277726 | 13387265 | 0.37 | 2.19 | -1.42 | 1.13 | 2.56 | 0.00 | UP-REGULATED | NM_001030272 | aryl hydrocarbon receptor nuclear translocator-like |
| TCONS_00007069 | XLOC_003143 | ASB13 | chr10 | 5638856 | 5666595 | 12.37 | 2.48 | 3.63 | 1.31 | -2.32 | 0.00 | DOWN-REGULATED | NM_024701 | ankyrin repeat and SOCS box-containing 13 |
| TCONS_00050743 | XLOC_022296 | ATF6B | chr6 | 32115267 | 32128240 | 1.28 | 4.99 | 0.36 | 2.32 | 1.96 | 0.01 | UP-REGULATED | NM_001136153_1 | activating transcription factor 6 beta |
| TCONS_00058829 | XLOC_025854 | ATP6V1H | chr8 | 53715542 | 53843311 | 1.60 | 5.01 | 0.68 | 2.33 | 1.65 | 0.01 | UP-REGULATED | NM_213619 | ATPase, H+ transporting, lysosomal 50/57kDa, V1 subunit H |
| TCONS_00063747 | XLOC_028093 | ATRX | chrX | 77504877 | 77786269 | 2.51 | 0.45 | 1.33 | -1.16 | -2.48 | 0.00 | DOWN-REGULATED | NM_138270 | alpha thalassemia/mental retardation syndrome X-linked (RAD54 homolog, S. cerevisiae) |
| TCONS_00036121 | XLOC_015734 | BAZ2B | chr2 | 159318978 | 159618042 | 0.33 | 1.48 | -1.60 | 0.56 | 2.16 | 0.00 | UP-REGULATED | NM_001289975 | bromodomain adjacent to zinc finger domain, 2B |
| TCONS_00037828 | XLOC_016486 | BFSP1 | chr20 | 17493904 | 17569220 | 3.03 | 0.23 | 1.60 | -2.14 | -3.74 | 0.04 | DOWN-REGULATED | NM_001278607 | beaded filament structural protein 1, filensin |
| TCONS_00040750 | XLOC_017799 | BRPF1 | chr3 | 9731749 | 9748015 | 3.18 | 1.03 | 1.67 | 0.05 | -1.62 | 0.00 | DOWN-REGULATED | NM_001003694 | bromodomain and PHD finger containing, 1 |
| TCONS_00010796 | XLOC_004804 | C11orf95 | chr11 | 63759891 | 63768641 | 7.25 | 1.65 | 2.86 | 0.72 | -2.13 | 0.00 | DOWN-REGULATED | NM_001144936 | hypothetical protein LOC65998 |
| TCONS_00039008 | XLOC_017031 | CBR3-AS1 | chr21 | 36131766 | 36156308 | 0.19 | 0.78 | -2.43 | -0.37 | 2.06 | 0.03 | UP-REGULATED | NR_038893 | CBR3 antisense RNA 1(CBR3-AS1) |
| TCONS_00061707 | XLOC_027175 | CCBL1 | chr9 | 128832941 | 128882075 | 0.24 | 1.55 | -2.05 | 0.63 | 2.68 | 0.01 | UP-REGULATED | NR_109829 | cysteine conjugate-beta lyase, cytoplasmic |
| TCONS_00026974 | XLOC_012138 | CCDC68 | chr18 | 54901508 | 54959508 | 0.28 | 1.30 | -1.84 | 0.38 | 2.22 | 0.02 | UP-REGULATED | NM_001143829 | coiled-coil domain containing 68 |
| TCONS_00045207 | XLOC_019827 | CCKAR | chr4 | 26481395 | 26490420 | 1.23 | 0.35 | 0.29 | -1.53 | -1.82 | 0.00 | DOWN-REGULATED | NM_000730 | cholecystokinin A receptor |
| TCONS_00036020 | XLOC_015698 | CCNT2-AS1 | chr2 | 134838615 | 134918606 | 0.89 | 4.47 | -0.17 | 2.16 | 2.33 | 0.00 | UP-REGULATED | NR_036549 | CCNT2 antisense RNA 1(CCNT2-AS1) |
| TCONS_00029235 | XLOC_013245 | CDKN2D | chr19 | 10566461 | 10568979 | 3.56 | 15.87 | 1.83 | 3.99 | 2.16 | 0.00 | UP-REGULATED | NM_079421 | cyclin-dependent kinase inhibitor 2D (p19, inhibits CDK4) |
| TCONS_00030132 | XLOC_013670 | CGB7 | chr19 | 49054273 | 49055740 | 0.43 | 1.28 | -1.23 | 0.36 | 1.59 | 0.02 | UP-REGULATED | NM_033142 | chorionic gonadotropin, beta polypeptide 7 |
| TCONS_00016294 | XLOC_007325 | CGRRF1 | chr14 | 54509868 | 54538616 | 9.43 | 3.28 | 3.24 | 1.72 | -1.52 | 0.00 | DOWN-REGULATED | NM_006568 | cell growth regulator with ring finger domain 1 |
| TCONS_00023982 | XLOC_010823 | CHMP6 | chr17 | 80991840 | 81000133 | 14.44 | 4.23 | 3.85 | 2.08 | -1.77 | 0.00 | DOWN-REGULATED | NM_024591 | chromatin modifying protein 6 |
| TCONS_00055491 | XLOC_024270 | CLDN12 | chr7 | 90403333 | 90415954 | 2.03 | 0.56 | 1.02 | -0.84 | -1.86 | 0.04 | DOWN-REGULATED | NM_001185073 | claudin 12 |
| TCONS_00011939 | XLOC_005295 | COPS7A | chr12 | 6723983 | 6731875 | 8.99 | 25.51 | 3.17 | 4.67 | 1.50 | 0.00 | UP-REGULATED | NM_001164094 | COP9 constitutive photomorphogenic homolog subunit 7A (Arabidopsis) |
| TCONS_00059956 | XLOC_026348 | DCAF10 | chr9 | 37800553 | 37867668 | 0.11 | 0.91 | -3.17 | -0.14 | 3.03 | 0.01 | UP-REGULATED | NM_001286810 | WD repeat domain 32 |
| TCONS_00008796 | XLOC_003865 | DGKZ | chr11 | 46332904 | 46380554 | 1.05 | 4.29 | 0.07 | 2.10 | 2.03 | 0.05 | UP-REGULATED | NM_001199268 | diacylglycerol kinase, zeta 104kDa |
| TCONS_00022559 | XLOC_010193 | DPH1 | chr17 | 2030136 | 2043431 | 20.94 | 7.40 | 4.39 | 2.89 | -1.50 | 0.01 | DOWN-REGULATED | NM_001383 | DPH1 homolog (S. cerevisiae) |
| TCONS_00005433 | XLOC_002392 | DSTYK | chr1 | 205142502 | 205211599 | 0.75 | 0.09 | -0.42 | -3.43 | -3.01 | 0.00 | DOWN-REGULATED | NM_015375 | dual serine/threonine and tyrosine protein kinase |
| TCONS_00003380 | XLOC_001489 | ECE1 | chr1 | 21217246 | 21345541 | 0.54 | 1.96 | -0.89 | 0.97 | 1.86 | 0.04 | UP-REGULATED | NM_001113349 | endothelin converting enzyme 1 |
| TCONS_00035587 | XLOC_015485 | EGR4 | chr2 | 73290928 | 73293701 | 0.14 | 0.69 | -2.82 | -0.54 | 2.28 | 0.00 | UP-REGULATED | NM_001965 | early growth response 4 |
| TCONS_00036626 | XLOC_015946 | EPHA4 | chr2 | 221418026 | 221574202 | 0.34 | 1.39 | -1.57 | 0.48 | 2.05 | 0.02 | UP-REGULATED | NM_001304536 | EPH receptor A4 |
| TCONS_00000746 | XLOC_000342 | EXO5 | chr1 | 40508760 | 40516542 | 3.40 | 0.58 | 1.76 | -0.80 | -2.56 | 0.00 | DOWN-REGULATED | NM_022774 | exonuclease 5(EXO5) |
| TCONS_00035494 | XLOC_015429 | FAM161A | chr2 | 61824847 | 61854143 | 0.30 | 1.04 | -1.72 | 0.06 | 1.78 | 0.01 | UP-REGULATED | NR_037710 | family with sequence similarity 161, member A |
| TCONS_00006970 | XLOC_003087 | FAM175B | chr10 | 124801784 | 124836670 | 9.94 | 1.53 | 3.31 | 0.61 | -2.70 | 0.00 | DOWN-REGULATED | NM_032182 | family with sequence similarity 175, member B |
| TCONS_00037533 | XLOC_016348 | FAM217B | chr20 | 59933763 | 59948647 | 8.11 | 1.96 | 3.02 | 0.97 | -2.05 | 0.00 | DOWN-REGULATED | NM_022106 | |
| TCONS_00004631 | XLOC_002042 | FAM63A | chr1 | 150996824 | 151008378 | 1.49 | 0.42 | 0.57 | -1.25 | -1.82 | 0.02 | DOWN-REGULATED | NM_001163258 | family with sequence similarity 63, member A |
| TCONS_00018521 | XLOC_008376 | FEM1B | chr15 | 68277802 | 68295865 | 8.54 | 2.38 | 3.09 | 1.25 | -1.84 | 0.00 | DOWN-REGULATED | NM_015322 | fem-1 homolog b (C. elegans) |
| TCONS_00060820 | XLOC_026738 | FOXD4 | chr9 | 116230 | 118417 | 0.26 | 0.90 | -1.97 | -0.15 | 1.82 | 0.00 | UP-REGULATED | NM_207305 | forkhead box D4 |
| TCONS_00025548 | XLOC_011545 | FOXJ1 | chr17 | 76136333 | 76240309 | 1.72 | 0.57 | 0.79 | -0.82 | -1.60 | 0.03 | DOWN-REGULATED | NM_001454 | forkhead box J1 |
| TCONS_00050992 | XLOC_022387 | FRS3 | chr6 | 41770175 | 41779905 | 1.47 | 0.45 | 0.55 | -1.16 | -1.71 | 0.00 | DOWN-REGULATED | NM_006653 | fibroblast growth factor receptor substrate 3 |
| TCONS_00042637 | XLOC_018631 | FYCO1 | chr3 | 45917898 | 45995824 | 1.30 | 0.39 | 0.37 | -1.36 | -1.74 | 0.00 | DOWN-REGULATED | NM_024513 | FYVE and coiled-coil domain containing 1 |
| TCONS_00055497 | XLOC_024272 | FZD1 | chr7 | 91264467 | 91268817 | 0.64 | 0.17 | -0.64 | -2.53 | -1.89 | 0.00 | DOWN-REGULATED | NM_003505 | frizzled homolog 1 (Drosophila) |
| TCONS_00044798 | XLOC_019630 | GAB1 | chr4 | 143336829 | 143474565 | 2.08 | 0.71 | 1.05 | -0.50 | -1.56 | 0.00 | DOWN-REGULATED | NM_002039 | GRB2-associated binding protein 1 |
| TCONS_00019227 | XLOC_008708 | GABPB1 | chr15 | 50277191 | 50358306 | 8.10 | 1.96 | 3.02 | 0.97 | -2.05 | 0.00 | DOWN-REGULATED | NM_016654 | GA binding protein transcription factor, beta subunit 1 |
| TCONS_00023847 | XLOC_010778 | GALR2 | chr17 | 76074800 | 76077541 | 0.67 | 6.05 | -0.57 | 2.60 | 3.17 | 0.00 | UP-REGULATED | NM_003857 | galanin receptor 2 |
| TCONS_00028261 | XLOC_012744 | GEMIN7 | chr19 | 45079259 | 45091524 | 8.25 | 2.71 | 3.04 | 1.44 | -1.61 | 0.00 | DOWN-REGULATED | NM_024707 | gem (nuclear organelle) associated protein 7 |
| TCONS_00004160 | XLOC_001838 | GEMIN8P4 | chr1 | 89993264 | 89994966 | 0.85 | 2.43 | -0.23 | 1.28 | 1.51 | 0.00 | UP-REGULATED | NR_002830 | FAM51A1 pseudogene |
| TCONS_00016127 | XLOC_007253 | GMPR2 | chr14 | 24232421 | 24239241 | 3.20 | 9.45 | 1.68 | 3.24 | 1.56 | 0.02 | UP-REGULATED | NM_001002001 | guanosine monophosphate reductase 2 |
| TCONS_00006713 | XLOC_002962 | GOLGA7B | chr10 | 97850237 | 98030828 | 0.25 | 0.98 | -1.98 | -0.04 | 1.95 | 0.00 | UP-REGULATED | NM_001010917 | golgi autoantigen, golgin subfamily a, 7B |
| TCONS_00043580 | XLOC_019054 | GOLIM4 | chr3 | 168008672 | 168095925 | 0.69 | 2.29 | -0.55 | 1.19 | 1.74 | 0.02 | UP-REGULATED | NM_001308155 | golgi integral membrane protein 4 |
| TCONS_00027677 | XLOC_012468 | GTPBP3 | chr19 | 17334981 | 17342731 | 2.98 | 1.02 | 1.58 | 0.03 | -1.55 | 0.02 | DOWN-REGULATED | NM_001128855 | GTP binding protein 3 (mitochondrial) |
| TCONS_00042931 | XLOC_018757 | HESX1 | chr3 | 57197915 | 57200252 | 0.15 | 0.70 | -2.76 | -0.51 | 2.25 | 0.04 | UP-REGULATED | NM_003865 | HESX homeobox 1 |
| TCONS_00003703 | XLOC_001639 | HEYL | chr1 | 39623430 | 39639676 | 0.74 | 0.24 | -0.43 | -2.08 | -1.65 | 0.00 | DOWN-REGULATED | NM_014571 | hairy/enhancer-of-split related with YRPW motif-like |
| TCONS_00005941 | XLOC_002654 | HSPA14 | chr10 | 14838159 | 14871741 | 1.89 | 6.14 | 0.92 | 2.62 | 1.70 | 0.02 | UP-REGULATED | NM_001278205 | heat shock 70kDa protein 14 |
| TCONS_00049330 | XLOC_021637 | HSPA1A | chr6 | 31815513 | 31817942 | 24.82 | 132.28 | 4.63 | 7.05 | 2.41 | 0.00 | UP-REGULATED | NM_005345_3 | heat shock 70kDa protein 1A; heat shock 70kDa protein 1B |
| TCONS_00049331 | XLOC_021638 | HSPA1B | chr6 | 31827734 | 31830254 | 21.94 | 71.69 | 4.46 | 6.16 | 1.71 | 0.00 | UP-REGULATED | NM_005346 | heat shock protein family A (Hsp70) member 1B(HSPA1B) |
| TCONS_00050724 | XLOC_022285 | HSPA1L | chr6 | 31809618 | 31815058 | 0.69 | 2.68 | -0.54 | 1.42 | 1.97 | 0.00 | UP-REGULATED | NM_005527_3 | heat shock 70kDa protein 1-like |
| TCONS_00002130 | XLOC_000921 | HSPA6 | chr1 | 161524539 | 161526897 | 0.45 | 4.91 | -1.14 | 2.30 | 3.44 | 0.00 | UP-REGULATED | NM_002155 | heat shock 70kDa protein 7 (HSP70B); heat shock 70kDa protein 6 (HSP70B') |
| TCONS_00006587 | XLOC_002912 | IFIT5 | chr10 | 89414567 | 89421002 | 0.71 | 2.11 | -0.50 | 1.08 | 1.58 | 0.00 | UP-REGULATED | NM_012420 | interferon-induced protein with tetratricopeptide repeats 5 |
| TCONS_00004996 | XLOC_002191 | IGSF9 | chr1 | 159927038 | 159945596 | 0.35 | 1.12 | -1.52 | 0.16 | 1.68 | 0.03 | UP-REGULATED | NM_020789 | immunoglobulin superfamily, member 9 |
| TCONS_00028312 | XLOC_012775 | INAFM1 | chr19 | 47274401 | 47275723 | 1.45 | 9.17 | 0.54 | 3.20 | 2.66 | 0.00 | UP-REGULATED | NM_178511 | InaF motif containing 1(INAFM1) |
| TCONS_00056175 | XLOC_024573 | INSIG1 | chr7 | 155297775 | 155310235 | 6.11 | 17.60 | 2.61 | 4.14 | 1.53 | 0.01 | UP-REGULATED | NM_198336 | insulin induced gene 1 |
| TCONS_00035758 | XLOC_015564 | KANSL3 | chr2 | 96593154 | 96638379 | 5.61 | 1.23 | 2.49 | 0.30 | -2.19 | 0.04 | DOWN-REGULATED | NM_001115016 | KAT8 regulatory NSL complex subunit 3(KANSL3) |
| TCONS_00015597 | XLOC_006973 | KBTBD7 | chr13 | 41132927 | 41263577 | 2.74 | 0.43 | 1.45 | -1.23 | -2.68 | 0.00 | DOWN-REGULATED | NM_032138 | kelch repeat and BTB (POZ) domain containing 7 |
| TCONS_00041387 | XLOC_018046 | KCTD6 | chr3 | 58492095 | 58502360 | 9.45 | 2.40 | 3.24 | 1.26 | -1.98 | 0.00 | DOWN-REGULATED | NM_001128214 | potassium channel tetramerisation domain containing 6 |
| TCONS_00020569 | XLOC_009282 | KDM8 | chr16 | 27203485 | 27221768 | 1.94 | 0.63 | 0.96 | -0.67 | -1.62 | 0.00 | DOWN-REGULATED | NM_024773 | lysine demethylase 8(KDM8) |
| TCONS_00029745 | XLOC_013486 | LGALS4 | chr19 | 38801670 | 38813100 | 1.27 | 6.52 | 0.34 | 2.70 | 2.36 | 0.00 | UP-REGULATED | NM_006149 | lectin, galactoside-binding, soluble, 4 |
| TCONS_00003018 | XLOC_001318 | LINC00115 | chr1 | 826205 | 827522 | 0.28 | 1.22 | -1.83 | 0.28 | 2.12 | 0.01 | UP-REGULATED | NR_024321 | |
| TCONS_00029560 | XLOC_013409 | LINC00662 | chr19 | 27790492 | 27806780 | 0.89 | 0.30 | -0.16 | -1.72 | -1.56 | 0.02 | DOWN-REGULATED | NR_027301 | long intergenic non-protein coding RNA 662(LINC00662) |
| TCONS_00048600 | XLOC_021312 | LOC100268168 | chr5 | 172954782 | 172959368 | 0.18 | 0.59 | -2.44 | -0.75 | 1.69 | 0.03 | UP-REGULATED | NR_026682 | hypothetical LOC100268168 |
| TCONS_00036852 | XLOC_016051 | LOC388780 | chr20 | 2206927 | 2213151 | 0.34 | 1.03 | -1.57 | 0.04 | 1.61 | 0.04 | UP-REGULATED | NM_001287682 | hypothetical LOC388780 |
| TCONS_00039203 | XLOC_017111 | LSS | chr21 | 46188445 | 46228824 | 5.91 | 18.72 | 2.56 | 4.23 | 1.66 | 0.05 | UP-REGULATED | NM_001145436 | lanosterol synthase (2,3-oxidosqualene-lanosterol cyclase) |
| TCONS_00004639 | XLOC_002045 | LYSMD1 | chr1 | 151156628 | 151176071 | 1.91 | 9.04 | 0.94 | 3.18 | 2.24 | 0.00 | UP-REGULATED | NM_212551 | LysM, putative peptidoglycan-binding, domain containing 1 |
| TCONS_00050272 | XLOC_022074 | MAP3K4 | chr6 | 160991726 | 161117385 | 1.79 | 0.58 | 0.84 | -0.78 | -1.61 | 0.00 | DOWN-REGULATED | NM_006724 | mitogen-activated protein kinase kinase kinase 4 |
| TCONS_00047973 | XLOC_021051 | MBLAC2 | chr5 | 90458202 | 90474768 | 0.96 | 0.27 | -0.06 | -1.89 | -1.83 | 0.00 | DOWN-REGULATED | NM_203406 | metallo-beta-lactamase domain containing 2 |
| TCONS_00041951 | XLOC_018325 | MBNL1 | chr3 | 152262615 | 152465780 | 0.54 | 1.99 | -0.90 | 0.99 | 1.90 | 0.04 | UP-REGULATED | NM_207295 | muscleblind-like (Drosophila) |
| TCONS_00051472 | XLOC_022594 | MCM9 | chr6 | 118813448 | 118935162 | 2.36 | 0.47 | 1.24 | -1.10 | -2.34 | 0.05 | DOWN-REGULATED | NM_153255 | minichromosome maintenance complex component 9 |
| TCONS_00014170 | XLOC_006277 | MDM1 | chr12 | 68294565 | 68332381 | 0.42 | 1.33 | -1.25 | 0.41 | 1.66 | 0.04 | UP-REGULATED | NM_001205028 | Mdm1 nuclear protein homolog (mouse) |
| TCONS_00010013 | XLOC_004424 | MIR210HG | chr11 | 565656 | 568457 | 0.80 | 3.71 | -0.33 | 1.89 | 2.22 | 0.00 | UP-REGULATED | NR_038262 | MIR210 host gene(MIR210HG) |
| TCONS_00028997 | XLOC_013106 | MOB3A | chr19 | 2071035 | 2096270 | 2.75 | 0.88 | 1.46 | -0.19 | -1.65 | 0.00 | DOWN-REGULATED | NM_130807 | MOB kinase activator 3A(MOB3A) |
| TCONS_00044933 | XLOC_019687 | MSMO1 | chr4 | 165327665 | 165343162 | 10.48 | 47.72 | 3.39 | 5.58 | 2.19 | 0.02 | UP-REGULATED | NM_001017369 | methylsterol monooxygenase 1(MSMO1) |
| TCONS_00000369 | XLOC_000180 | NBPF3 | chr1 | 21440089 | 21484900 | 0.48 | 2.43 | -1.07 | 1.28 | 2.34 | 0.00 | UP-REGULATED | NM_001256417 | neuroblastoma breakpoint family, member 3 |
| TCONS_00001581 | XLOC_000703 | NBPF8 | chr1 | 120436350 | 120467844 | 0.27 | 1.32 | -1.89 | 0.40 | 2.29 | 0.01 | UP-REGULATED | NR_102404 | neuroblastoma breakpoint family member 8(NBPF8) |
| TCONS_00057494 | XLOC_025177 | NCAPG2 | chr7 | 158631168 | 158704831 | 0.91 | 3.13 | -0.14 | 1.65 | 1.79 | 0.03 | UP-REGULATED | NM_001281932 | non-SMC condensin II complex, subunit G2 |

| | | | | | | | | | | | | | | |
|---|---|---|---|---|---|---|---|---|---|---|---|---|---|---|
| TCONS_00060625 | XLOC_026638 | NCS1 | chr9 | 130172577 | 130237304 | 4.36 | 1.26 | 2.13 | 0.33 | -1.79 | 0.01 | DOWN-REGULATED | NM_014286 | frequenin homolog (Drosophila) |
| TCONS_00060626 | XLOC_026638 | NCS1 | chr9 | 130172577 | 130237304 | 0.63 | 2.93 | -0.67 | 1.55 | 2.22 | 0.00 | UP-REGULATED | NM_001128826 | frequenin homolog (Drosophila) |
| TCONS_00013966 | XLOC_006189 | NFE2 | chr12 | 54292106 | 54301037 | 3.05 | 0.88 | 1.61 | -0.19 | -1.80 | 0.01 | DOWN-REGULATED | NM_001136023 | nuclear factor (erythroid-derived 2), 45kDa |
| TCONS_00042785 | XLOC_018687 | NICN1 | chr3 | 49416777 | 49429324 | 1.04 | 0.11 | 0.06 | -3.14 | -3.20 | 0.04 | DOWN-REGULATED | NM_032316 | nicolin 1 |
| TCONS_00028303 | XLOC_012771 | NPAS1 | chr19 | 47020885 | 47045759 | 0.32 | 1.05 | -1.63 | 0.08 | 1.71 | 0.00 | UP-REGULATED | NM_002517 | neuronal PAS domain protein 1 |
| TCONS_00012430 | XLOC_005489 | NR4A1 | chr12 | 52022831 | 52059507 | 3.26 | 10.01 | 1.71 | 3.32 | 1.62 | 0.00 | UP-REGULATED | NM_173157 | nuclear receptor subfamily 4, group A, member 1 |
| TCONS_00009133 | XLOC_004027 | NUDT22 | chr11 | 64226257 | 64230016 | 15.76 | 4.45 | 3.98 | 2.16 | -1.82 | 0.00 | DOWN-REGULATED | NM_032344 | nudix (nucleoside diphosphate linked moiety X)-type motif 22 |
| TCONS_00047774 | XLOC_020962 | PDE4D | chr5 | 58969040 | 60547657 | 4.66 | 0.86 | 2.22 | -0.22 | -2.44 | 0.00 | DOWN-REGULATED | NM_001197222 | phosphodiesterase 4D, cAMP-specific (phosphodiesterase E3 dunce homolog, Drosophila) |
| TCONS_00037002 | XLOC_016114 | PET117 | chr20 | 18137854 | 18188387 | 41.08 | 14.32 | 5.36 | 3.84 | -1.52 | 0.00 | DOWN-REGULATED | NM_001164811 | PET117 homolog(PET117) |
| TCONS_00004505 | XLOC_001991 | PEX11B | chr1 | 145911347 | 145918924 | 10.69 | 3.48 | 3.42 | 1.80 | -1.62 | 0.00 | DOWN-REGULATED | NM_003846 | peroxisomal biogenesis factor 11 beta |
| TCONS_00033588 | XLOC_014584 | PEX13 | chr2 | 60940412 | 61051990 | 10.70 | 2.60 | 3.42 | 1.38 | -2.04 | 0.00 | DOWN-REGULATED | NM_002618 | peroxisomal biogenesis factor 13 |
| TCONS_00014714 | XLOC_006488 | PITPNM2 | chr12 | 122983479 | 123110489 | 0.71 | 0.25 | -0.50 | -2.00 | -1.50 | 0.04 | DOWN-REGULATED | NM_020845 | phosphatidylinositol transfer protein, membrane-associated 2 |
| TCONS_00040295 | XLOC_017587 | PLA2G6 | chr22 | 38111494 | 38181829 | 0.26 | 1.16 | -1.95 | 0.21 | 2.16 | 0.01 | UP-REGULATED | NM_001199562 | phospholipase A2, group VI (cytosolic, calcium-independent) |
| TCONS_00037942 | XLOC_016551 | PLAGL2 | chr20 | 32192503 | 32207743 | 11.73 | 2.86 | 3.55 | 1.52 | -2.04 | 0.00 | DOWN-REGULATED | NM_002657 | pleiomorphic adenoma gene-like 2; similar to pleiomorphic adenoma gene-like 2 |
| TCONS_00005896 | XLOC_002629 | PRKCQ-AS1 | chr10 | 6580424 | 6585361 | 1.23 | 0.23 | 0.30 | -2.09 | -2.39 | 0.00 | DOWN-REGULATED | NR_036502 | |
| TCONS_00005897 | XLOC_002629 | PRKCQ-AS1 | chr10 | 6580424 | 6585361 | 1.40 | 0.35 | 0.48 | -1.51 | -1.99 | 0.00 | DOWN-REGULATED | NR_036503 | |
| TCONS_00001395 | XLOC_000613 | PRMT6 | chr1 | 107056644 | 107059296 | 10.13 | 2.56 | 3.34 | 1.36 | -1.98 | 0.00 | DOWN-REGULATED | NM_018137 | protein arginine methyltransferase 6 |
| TCONS_00041163 | XLOC_017964 | PTPN23 | chr3 | 47380981 | 47413441 | 0.89 | 4.05 | -0.16 | 2.02 | 2.18 | 0.03 | UP-REGULATED | NM_001304482 | protein tyrosine phosphatase, non-receptor type 23 |
| TCONS_00047601 | XLOC_020881 | RAD1 | chr5 | 34905260 | 34915675 | 2.72 | 0.52 | 1.44 | -0.94 | -2.38 | 0.00 | DOWN-REGULATED | NR_026591 | RAD1 homolog (S. pombe) |
| TCONS_00013290 | XLOC_005891 | RAD52 | chr12 | 911735 | 990041 | 3.35 | 0.63 | 1.75 | -0.67 | -2.42 | 0.00 | DOWN-REGULATED | NM_134424 | RAD52 homolog (S. cerevisiae) |
| TCONS_00061757 | XLOC_027199 | RALGDS | chr9 | 133097719 | 133149220 | 0.56 | 3.11 | -0.82 | 1.64 | 2.46 | 0.00 | UP-REGULATED | NM_001271774 | ral guanine nucleotide dissociation stimulator |
| TCONS_00038045 | XLOC_016592 | RBL1 | chr20 | 36996350 | 37096000 | 4.83 | 1.58 | 2.27 | 0.66 | -1.62 | 0.00 | DOWN-REGULATED | NM_002895 | retinoblastoma-like 1 (p107) |
| TCONS_00041226 | XLOC_017989 | RBM5 | chr3 | 50088907 | 50118964 | 8.74 | 2.99 | 3.13 | 1.58 | -1.55 | 0.04 | DOWN-REGULATED | NR_036627 | RNA binding motif protein 5 |
| TCONS_00030479 | XLOC_013805 | RDH13 | chr19 | 55044323 | 55069546 | 1.56 | 4.92 | 0.64 | 2.30 | 1.65 | 0.01 | UP-REGULATED | NM_138412_7 | retinol dehydrogenase 13 (all-trans/9-cis) |
| TCONS_00063691 | XLOC_028064 | RGAG4 | chrX | 71911087 | 72143574 | 0.60 | 0.20 | -0.75 | -2.32 | -1.57 | 0.00 | DOWN-REGULATED | NM_001024455 | retrotransposon gag domain containing 4 |
| TCONS_00005261 | XLOC_002315 | RGS16 | chr1 | 182598622 | 182604413 | 1.79 | 5.67 | 0.84 | 2.50 | 1.66 | 0.00 | UP-REGULATED | NM_002928 | regulator of G-protein signaling 16 |
| TCONS_00048025 | XLOC_021067 | RIOK2 | chr5 | 97160866 | 97183301 | 11.57 | 3.83 | 3.53 | 1.94 | -1.60 | 0.00 | DOWN-REGULATED | NM_018343 | RIO kinase 2 (yeast) |
| TCONS_00015450 | XLOC_006912 | RNF6 | chr13 | 26212767 | 26222371 | 4.19 | 1.26 | 2.07 | 0.34 | -1.73 | 0.02 | DOWN-REGULATED | NM_005977 | ring finger protein (C3H2C3 type) 6 |
| TCONS_00017051 | XLOC_007697 | SALL2 | chr14 | 21521080 | 21537216 | 1.46 | 0.41 | 0.55 | -1.27 | -1.82 | 0.00 | DOWN-REGULATED | NM_005407 | sal-like 2 (Drosophila) |
| TCONS_00022284 | XLOC_010063 | SDR42E1 | chr16 | 81997645 | 82011488 | 4.06 | 0.90 | 2.02 | -0.15 | -2.17 | 0.00 | DOWN-REGULATED | NM_145168 | short chain dehydrogenase/reductase family 42E, member 1 |
| TCONS_00026487 | XLOC_011933 | SKA1 | chr18 | 50375021 | 50394168 | 3.00 | 10.01 | 1.58 | 3.32 | 1.74 | 0.00 | UP-REGULATED | NM_145060 | chromosome 18 open reading frame 24 |
| TCONS_00041835 | XLOC_018269 | SLC35G2 | chr3 | 136819018 | 136862063 | 0.52 | 3.79 | -0.95 | 1.92 | 2.87 | 0.01 | UP-REGULATED | NM_001097599 | solute carrier family 35 member G2(SLC35G2) |
| TCONS_00041837 | XLOC_018269 | SLC35G2 | chr3 | 136819018 | 136862063 | 2.37 | 7.68 | 1.25 | 2.94 | 1.69 | 0.00 | UP-REGULATED | NM_025246 | solute carrier family 35 member G2(SLC35G2) |
| TCONS_00040448 | XLOC_017659 | SMC1B | chr22 | 45344063 | 45413619 | 0.17 | 0.83 | -2.52 | -0.28 | 2.25 | 0.02 | UP-REGULATED | NM_148674 | structural maintenance of chromosomes 1B |
| TCONS_00026718 | XLOC_012042 | SPIRE1 | chr18 | 12446511 | 12657913 | 0.21 | 1.00 | -2.28 | -0.01 | 2.27 | 0.02 | UP-REGULATED | NM_001128627 | spire homolog 1 (Drosophila) |
| TCONS_00003892 | XLOC_001704 | STIL | chr1 | 47250138 | 47314147 | 0.61 | 2.63 | -0.72 | 1.40 | 2.11 | 0.05 | UP-REGULATED | CUFF.111.7 | SCL/TAL1 interrupting locus |
| TCONS_00029341 | XLOC_013294 | STX10 | chr19 | 13144057 | 13150374 | 2.48 | 7.58 | 1.31 | 2.92 | 1.61 | 0.00 | UP-REGULATED | NM_001271610 | syntaxin 10 |
| TCONS_00056723 | XLOC_024831 | STX1A | chr7 | 73699204 | 73719687 | 1.17 | 3.63 | 0.23 | 1.86 | 1.63 | 0.00 | UP-REGULATED | NM_001165903 | syntaxin 1A (brain) |
| TCONS_00060307 | XLOC_026518 | TAL2 | chr9 | 105662456 | 105663104 | 0.60 | 1.74 | -0.73 | 0.80 | 1.53 | 0.04 | UP-REGULATED | NM_005421 | T-cell acute lymphocytic leukemia 2 |
| TCONS_00062682 | XLOC_027635 | TCEAL3 | chrX | 103607905 | 103609927 | 2.36 | 8.93 | 1.24 | 3.16 | 1.92 | 0.00 | UP-REGULATED | NM_032926 | transcription elongation factor A (SII)-like 3 |
| TCONS_00012967 | XLOC_005749 | TCHP | chr12 | 109900273 | 109918069 | 3.27 | 1.10 | 1.71 | 0.14 | -1.57 | 0.00 | DOWN-REGULATED | NM_001143852 | trichoplein, keratin filament binding |
| TCONS_00024036 | XLOC_010846 | TEX19 | chr17 | 82359246 | 82363776 | 3.95 | 0.99 | 1.98 | -0.01 | -1.99 | 0.00 | DOWN-REGULATED | NM_207459 | testis expressed 19 |
| TCONS_00026208 | XLOC_011817 | TGIF1 | chr18 | 3411926 | 3458408 | 1.69 | 7.31 | 0.76 | 2.87 | 2.11 | 0.01 | UP-REGULATED | NM_173208 | TGFB-induced factor homeobox 1 |
| TCONS_00005608 | XLOC_002462 | TLR5 | chr1 | 223109405 | 223143282 | 0.22 | 0.89 | -2.16 | -0.18 | 1.99 | 0.00 | UP-REGULATED | NM_003268 | toll-like receptor 5 |
| TCONS_00043343 | XLOC_018937 | TMCC1 | chr3 | 129647791 | 129893576 | 1.29 | 5.20 | 0.37 | 2.38 | 2.00 | 0.00 | UP-REGULATED | NM_001128224 | transmembrane and coiled-coil domain family 1 |
| TCONS_00009255 | XLOC_004084 | TMEM151A | chr11 | 66291901 | 66296664 | 0.53 | 3.16 | -0.92 | 1.66 | 2.58 | 0.00 | UP-REGULATED | NM_153266 | transmembrane protein 151A |
| TCONS_00009798 | XLOC_004318 | TMEM25 | chr11 | 118436489 | 118566076 | 5.44 | 1.58 | 2.44 | 0.66 | -1.78 | 0.03 | DOWN-REGULATED | NM_032780 | transmembrane protein 25 |
| TCONS_00001990 | XLOC_000864 | TMEM79 | chr1 | 156282912 | 156292443 | 2.71 | 8.82 | 1.44 | 3.14 | 1.70 | 0.00 | UP-REGULATED | NR_026678 | transmembrane protein 79 |
| TCONS_00058633 | XLOC_025760 | TNFRSF10D | chr8 | 23135590 | 23164030 | 11.71 | 35.10 | 3.55 | 5.13 | 1.58 | 0.00 | UP-REGULATED | NM_003840 | tumor necrosis factor receptor superfamily, member 10d, decoy with truncated death domain |
| TCONS_00015090 | XLOC_006714 | TRIM13 | chr13 | 49982551 | 50528643 | 2.58 | 0.85 | 1.36 | -0.24 | -1.61 | 0.03 | DOWN-REGULATED | CUFF.963.4 | tripartite motif-containing 13 |
| TCONS_00017636 | XLOC_007955 | TRIP11 | chr14 | 91967898 | 92040059 | 3.70 | 1.08 | 1.89 | 0.12 | -1.77 | 0.00 | DOWN-REGULATED | NM_004239 | hypothetical LOC341378; thyroid hormone receptor interactor 11 |
| TCONS_00063885 | XLOC_028161 | TSC22D3 | chrX | 107713221 | 107775787 | 0.25 | 0.87 | -2.02 | -0.20 | 1.82 | 0.05 | UP-REGULATED | NM_004089 | TSC22 domain family, member 3; GRAM domain containing 4 |
| TCONS_00001014 | XLOC_000457 | TTC4 | chr1 | 54641739 | 54742655 | 9.26 | 2.50 | 3.21 | 1.32 | -1.89 | 0.00 | DOWN-REGULATED | NM_004623 | tetratricopeptide repeat domain 4 |
| TCONS_00016978 | XLOC_007672 | TTC5 | chr14 | 20289140 | 20305994 | 9.27 | 2.02 | 3.21 | 1.02 | -2.19 | 0.00 | DOWN-REGULATED | NM_138376 | tetratricopeptide repeat domain 5 |
| TCONS_00015929 | XLOC_007156 | TUBGCP3 | chr13 | 112485004 | 112588185 | 6.77 | 1.01 | 2.76 | 0.02 | -2.74 | 0.00 | DOWN-REGULATED | NM_006322 | tubulin, gamma complex associated protein 3 |
| TCONS_00025024 | XLOC_011307 | UBTF | chr17 | 44205032 | 44221626 | 6.52 | 1.45 | 2.70 | 0.54 | -2.17 | 0.04 | DOWN-REGULATED | CUFF.1538.5 | upstream binding transcription factor, RNA polymerase I |
| TCONS_00063958 | XLOC_028194 | UPF3B | chrX | 119834025 | 119853028 | 1.97 | 5.61 | 0.97 | 2.49 | 1.51 | 0.04 | UP-REGULATED | NM_080632 | UPF3 regulator of nonsense transcripts homolog B (yeast) |
| TCONS_00019410 | XLOC_008783 | VWA9 | chr15 | 65578757 | 65611289 | 5.21 | 17.91 | 2.38 | 4.16 | 1.78 | 0.03 | UP-REGULATED | NM_001207059 | von Willebrand factor A domain containing 9(VWA9) |
| TCONS_00020129 | XLOC_009080 | WFIKKN1 | chr16 | 631011 | 634116 | 0.12 | 0.82 | -3.08 | -0.29 | 2.79 | 0.01 | UP-REGULATED | NM_053284 | WAP, follistatin/kazal, immunoglobulin, kunitz and netrin domain containing 1 |
| TCONS_00058736 | XLOC_025822 | WHSC1L1 | chr8 | 38275042 | 38382272 | 0.92 | 0.30 | -0.13 | -1.72 | -1.59 | 0.03 | DOWN-REGULATED | NM_023034 | Wolf-Hirschhorn syndrome candidate 1-like 1 |
| TCONS_00011119 | XLOC_004931 | XRRA1 | chr11 | 74748867 | 74949187 | 1.64 | 4.85 | 0.71 | 2.28 | 1.56 | 0.01 | UP-REGULATED | NM_001270380 | X-ray radiation resistance associated 1 |
| TCONS_00014074 | XLOC_006227 | ZBTB39 | chr12 | 56998832 | 57006513 | 1.77 | 0.62 | 0.83 | -0.70 | -1.53 | 0.00 | DOWN-REGULATED | NM_014830 | zinc finger and BTB domain containing 39 |
| TCONS_00003941 | XLOC_001722 | ZCCHC11 | chr1 | 52423275 | 52553090 | 2.61 | 0.88 | 1.38 | -0.19 | -1.58 | 0.00 | DOWN-REGULATED | NM_001009881 | zinc finger, CCHC domain containing 11 |
| TCONS_00059380 | XLOC_026094 | ZFAT | chr8 | 134477787 | 134713049 | 0.28 | 1.37 | -1.82 | 0.46 | 2.27 | 0.01 | UP-REGULATED | NM_001174157 | zinc finger and AT hook domain containing |
| TCONS_00029668 | XLOC_013457 | ZFP82 | chr19 | 36391958 | 36418648 | 1.64 | 0.51 | 0.72 | -0.98 | -1.70 | 0.00 | DOWN-REGULATED | NM_133466 | zinc finger protein 82 homolog (mouse) |
| TCONS_00008959 | XLOC_003942 | ZFP91 | chr11 | 58579113 | 58625732 | 1.42 | 4.42 | 0.51 | 2.14 | 1.64 | 0.03 | UP-REGULATED | NM_001197051 | zinc finger protein 91 homolog (mouse); ZFP91-CNTF readthrough transcript; ciliary neurotrophic factor |
| TCONS_00037620 | XLOC_016390 | ZGPAT | chr20 | 63657809 | 63739107 | 20.31 | 6.24 | 4.34 | 2.64 | -1.70 | 0.00 | DOWN-REGULATED | NM_181485 | zinc finger, CCCH-type with G patch domain |
| TCONS_00049151 | XLOC_021569 | ZKSCAN3 | chr6 | 28349913 | 28369177 | 0.67 | 0.22 | -0.59 | -2.21 | -1.62 | 0.00 | DOWN-REGULATED | NM_024493 | zinc finger with KRAB and SCAN domains 3 |
| TCONS_00050583 | XLOC_022218 | ZKSCAN4 | chr6 | 28241704 | 28259252 | 1.18 | 0.28 | 0.23 | -1.82 | -2.06 | 0.00 | DOWN-REGULATED | NM_019110 | zinc finger with KRAB and SCAN domains 4 |
| TCONS_00049132 | XLOC_021563 | ZKSCAN8 | chr6 | 28141909 | 28159472 | 1.96 | 0.19 | 0.97 | -2.37 | -3.34 | 0.00 | DOWN-REGULATED | NM_006298 | zinc finger with KRAB and SCAN domains 8(ZKSCAN8) |
| TCONS_00003624 | XLOC_001603 | ZMYM6 | chr1 | 34986165 | 35031968 | 1.25 | 0.38 | 0.32 | -1.38 | -1.70 | 0.00 | DOWN-REGULATED | NM_007167 | hypothetical LOC100130633; zinc finger, MYM-type 6 |
| TCONS_00036571 | XLOC_015921 | ZNF142 | chr2 | 218637915 | 218659632 | 1.41 | 0.41 | 0.50 | -1.30 | -1.80 | 0.01 | DOWN-REGULATED | NM_001105537 | zinc finger protein 142 |
| TCONS_00027878 | XLOC_012567 | ZNF181 | chr19 | 34734574 | 34742869 | 2.26 | 0.67 | 1.18 | -0.58 | -1.76 | 0.00 | DOWN-REGULATED | NM_001029997 | zinc finger protein 181 |
| TCONS_00022188 | XLOC_010021 | ZNF19 | chr16 | 71474072 | 71489351 | 0.82 | 0.11 | -0.28 | -3.25 | -2.97 | 0.00 | DOWN-REGULATED | NM_006961 | zinc finger protein 19 |
| TCONS_00020292 | XLOC_009159 | ZNF205 | chr16 | 3110459 | 3120517 | 6.90 | 2.03 | 2.79 | 1.02 | -1.77 | 0.00 | DOWN-REGULATED | NM_001042428 | zinc finger protein 205 |
| TCONS_00027880 | XLOC_012568 | ZNF30 | chr19 | 34926902 | 34945172 | 2.01 | 0.19 | 1.00 | -2.43 | -3.43 | 0.00 | DOWN-REGULATED | NM_001099437 | zinc finger protein 30 |
| TCONS_00027862 | XLOC_012566 | ZNF302 | chr19 | 34677638 | 34686397 | 4.18 | 0.85 | 2.06 | -0.23 | -2.30 | 0.00 | DOWN-REGULATED | NM_001012320 | zinc finger protein 302 |
| TCONS_00050598 | XLOC_022226 | ZNF311 | chr6 | 28994816 | 29005260 | 0.72 | 0.21 | -0.47 | -2.23 | -1.76 | 0.00 | DOWN-REGULATED | NM_001010877_5 | zinc finger protein 311 |
| TCONS_00059531 | XLOC_026163 | ZNF34 | chr8 | 144772223 | 144787345 | 0.87 | 0.18 | -0.20 | -2.44 | -2.24 | 0.03 | DOWN-REGULATED | NM_001286769 | zinc finger protein 34 |
| TCONS_00057398 | XLOC_025135 | ZNF425 | chr7 | 149102785 | 149126346 | 0.80 | 0.25 | -0.33 | -1.98 | -1.65 | 0.00 | DOWN-REGULATED | NM_001001661 | zinc finger protein 425 |
| TCONS_00042620 | XLOC_018620 | ZNF445 | chr3 | 44439769 | 44477670 | 1.63 | 0.46 | 0.70 | -1.12 | -1.82 | 0.00 | DOWN-REGULATED | NM_181489 | zinc finger protein 445 |
| TCONS_00005801 | XLOC_002579 | ZNF496 | chr1 | 247300319 | 247331743 | 4.33 | 1.22 | 2.11 | 0.29 | -1.83 | 0.00 | DOWN-REGULATED | NM_032752 | zinc finger protein 496 |
| TCONS_00021620 | XLOC_009720 | ZNF500 | chr16 | 4750813 | 4767218 | 2.86 | 0.93 | 1.51 | -0.11 | -1.62 | 0.00 | DOWN-REGULATED | NM_021646 | zinc finger protein 500 |
| TCONS_00021931 | XLOC_009876 | ZNF629 | chr16 | 30778448 | 30787202 | 2.85 | 0.84 | 1.51 | -0.24 | -1.76 | 0.00 | DOWN-REGULATED | NM_001080417 | zinc finger protein 629 |
| TCONS_00039343 | XLOC_017162 | ZNF74 | chr22 | 20394114 | 20408463 | 2.27 | 0.73 | 1.18 | -0.46 | -1.64 | 0.00 | DOWN-REGULATED | NM_003426 | zinc finger protein 74 |
| TCONS_00049439 | XLOC_021688 | ZNF76 | chr6 | 35259713 | 35295987 | 0.78 | 2.83 | -0.36 | 1.50 | 1.86 | 0.00 | UP-REGULATED | NM_001292032 | zinc finger protein 76 (expressed in testis) |
| TCONS_00057399 | XLOC_025136 | ZNF777 | chr7 | 149431362 | 149460962 | 6.40 | 2.07 | 2.68 | 1.05 | -1.63 | 0.00 | DOWN-REGULATED | NM_015694 | zinc finger protein 777 |
| TCONS_00057397 | XLOC_025134 | ZNF786 | chr7 | 149069640 | 149090777 | 3.20 | 1.07 | 1.68 | 0.09 | -1.58 | 0.00 | DOWN-REGULATED | NM_152411 | zinc finger protein 786 |
| TCONS_00047430 | XLOC_020784 | ZNF879 | chr5 | 179023774 | 179034387 | 1.88 | 0.65 | 0.91 | -0.63 | -1.54 | 0.00 | DOWN-REGULATED | NM_001136116 | similar to hypothetical protein 9630041N07 |
| TCONS_00050591 | XLOC_022220 | ZSCAN12 | chr6 | 28378820 | 28399767 | 0.71 | 0.11 | -0.49 | -3.21 | -2.72 | 0.00 | DOWN-REGULATED | NR_028077 | zinc finger and SCAN domain containing 12 |
| TCONS_00049129 | XLOC_021562 | ZSCAN16 | chr6 | 28121794 | 28137316 | 5.27 | 1.15 | 2.40 | 0.20 | -2.20 | 0.00 | DOWN-REGULATED | NM_025231 | zinc finger and SCAN domain containing 16 |
| TCONS_00055579 | XLOC_024309 | ZSCAN25 | chr7 | 99616947 | 99632407 | 5.99 | 1.94 | 2.58 | 0.96 | -1.62 | 0.00 | DOWN-REGULATED | NM_145115 | zinc finger and SCAN domain containing 25(ZSCAN25) |

| Supplementary Table T3 : List of networks and genes involved identified by IPA analysis | | | |
|---|---|---|---|
| Network ID | Molecules in Network | Score | Focus Molecules |
| 1 | 26s Proteasome, ATF6B, ATP6V1H, ATRX, Ck2, EXO5, Fibrinogen, FOXJ1, Hdac, HISTONE,Histone h3,HSP,Hsp70,HSPA14,HSPA1A/HSPA1B,HSPA1L,HSPA6,NBPF10, (includes others), NBPF3, NFE2, NFkB (complex), NPAS1, RAD1, RAD52, RALGDS, RBM5,RNA polymerase II,RPA,TLR5,TNFRSF10D,TRIM13,TTC5,TUT4,UBTF,ZNF74 | 47 | 24 |
| 2 | ADRB,AGFG1,ANGPT1,CDKN2D,CG,CGB7,Collagen(s),Creb,cytochrome C, DGKZ, ECE1, EGR4,ERK1/2,FSH,FZD1,GAB1,Ikb,INSIG1,LDL,Lh,LSS,MAP2K1/2,Mek,MOB3A,MSMO1,NR 4A1,PDE4D,PDGF BB,PLA2G6,PLC gamma,Proinsulin,RGS16,TGIF1,TSC22D3,Vegf | 34 | 19 |
| 3 | AKR1A1,Akt,ARNTL,Calmodulin,CCDC68,CCKAR,E2f,EPHA4,estrogen receptor, FYCO1, GOLIM4, Gpcr, Growth hormone, HESX1, IgG1, Insulin, INTS14, MAP3K4, Mapk, MBNL1, N-cor, p85 (pik3r),PI3K (complex), Pkc(s),PRMT6, Rac, RAS, Rb, RBL1, Ubiquitin, ZFAT, ZKSCAN3, ZKSCAN4, ZNF496, ZSCAN12 | 32 | 18 |
| 4 | AMMECR1,BRD3,BTBD9,CAMKMT,CLDN12,DHRS3,DMKN,DSC2,ELAVL1,ELK4,FAM217B,FN BP1,HNRNPL,IFIT5,IGSF9,KANSL3,KCND1,KCTD6,MBNL2,miR-153-3p (miRNAs w/seed UGCAUAG),miR-4681 (miRNAs w/seed ACGGGAA), NCS1, PITPNM2, SKA1, SPIRE1, SRF, SRGAP1, TBC1D19,TUT4,USP24,ZBTB39,ZDHHC13,ZFP91,ZNF425,ZNF879 | 27 | 16 |
| 5 | ADGRB2,ANKRD37,ASB7,BFSP1,BFSP2,CEP152,CEP83,DSC2,FEM1B,GEMIN7,GPR37,GRHL 1,GTPBP3,HNF4A,KDM8,miR-181a-5p (and other miRNAs w/seed ACAUUCA), PET117, PLAGL2, PTAFR, RIOX1, SALL2, SGSH, SLC35G2, SMG5, SMG7, STARD10, STIL, TCHP, TGFB1,TRIP11,UPF3B,VIM,WFIKKN1,XPNPEP3,ZNF30 | 27 | 16 |
| 6 | ANKRD40,APP,ASB13,DNAJC5,F2,HEYL,KYAT1,LPAR6,MINDY1,MZT2A,N-type Calcium Channel, NAPB, PTCD2, RCBTB1, RIOK2, SLC25A38, SMTN, STX10, TMCC1, TRIP13, TUBGCP3, ULK4, UNC13B, VTN, ZKSCAN8, ZNF205, ZNF232, ZNF34, ZNF396, ZNF445, ZNF483, ZNF777, ZSCAN16, ZSCAN20, ZSCAN32 | 25 | 15 |
| 7 | ADCY2,ADRB2,ARMCX5,Beta Arrestin, CBX5, CCKAR, CHMP4A, CHMP6, CNBP, DNM1L, EGFR, Endophilin, G protein, GALR2, GMPR2, GNAT2, GNRHR, GPR37, KBTBD7, NPY1R, OXTR, PEX11B, Pik3r, Rgs, RXFP1, SNF8, TCEAL3, TM9SF3, TMEM25, TOMM70, XRRA1, ZFP82, ZNF302, ZNF483, ZSCAN25 | 21 | 13 |
| 8 | AURKA,BAG1,BAZ2B,BRPF1,CBR3-AS1, CCND1, CDH13, CDKN2D, CHMP5, DPH1, DSTYK, EPB41L1,FARP1,FOXD4,FZD7,GC-GCR dimer,GNPNAT1,Hspa1b,MBLAC2,MYCL,N-Cadherin, NFKBIA, NSD3, Ppp1cc, prostaglandin J2, PTPN23, PTPRU, PWWP2B, RNF219, RNF6, STAC3, TAL2,TNRC18,TRIM23,TTC4 | 21 | 13 |
