# Supplementary figures and images for: Zinc Chelation Specifically Inhibits Early Stages of Dengue Virus Replication by Activation of NF-κB and Induction of Antiviral Response in Epithelial Cells
Source: Front Immunol. 2019 Oct 1;10:2347. doi: 10.3389/fimmu.2019.02347 (PMC6779808; doi:10.3389/fimmu.2019.02347)

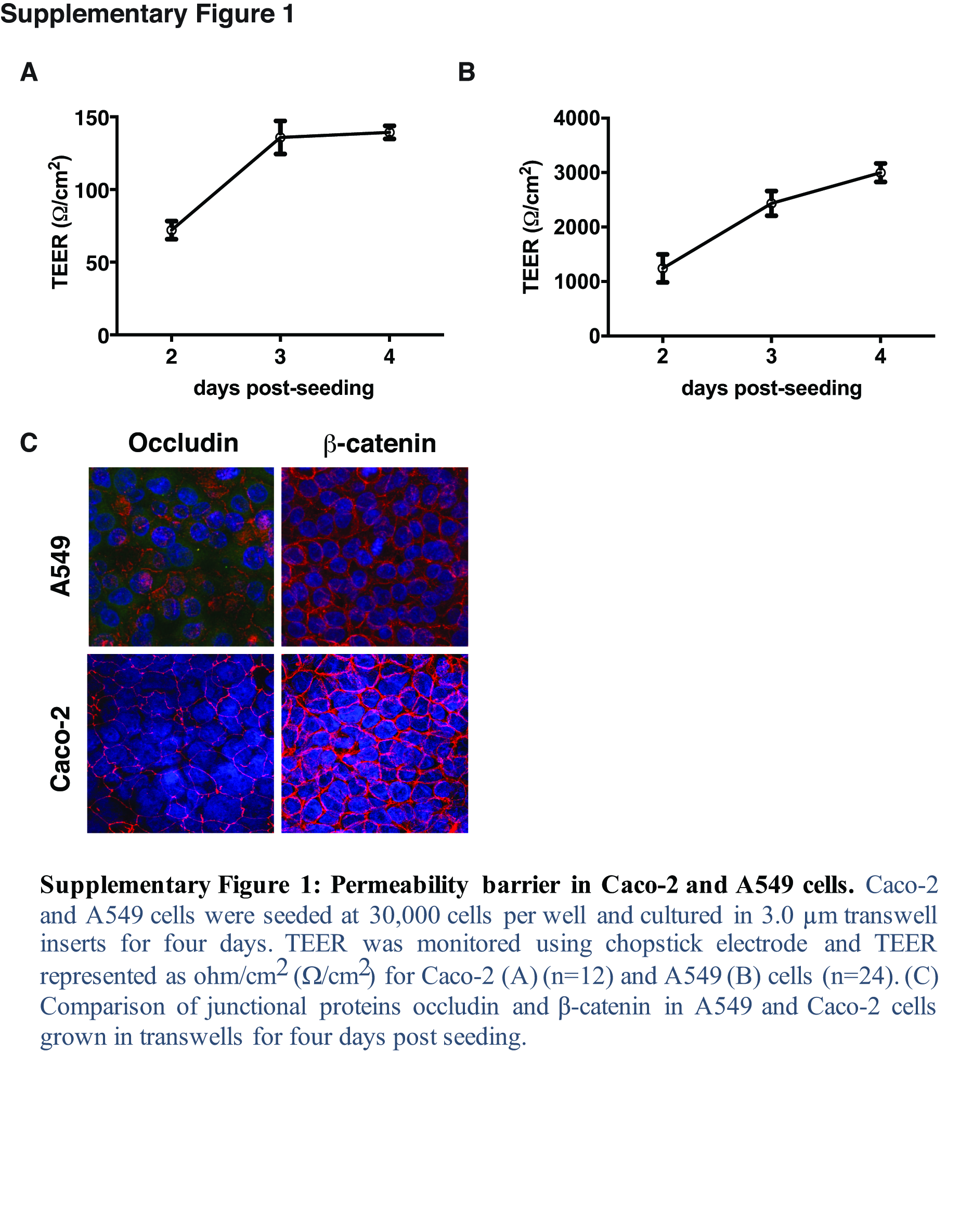

Supplement: Supplementary file 2 [file Image_1.TIF]

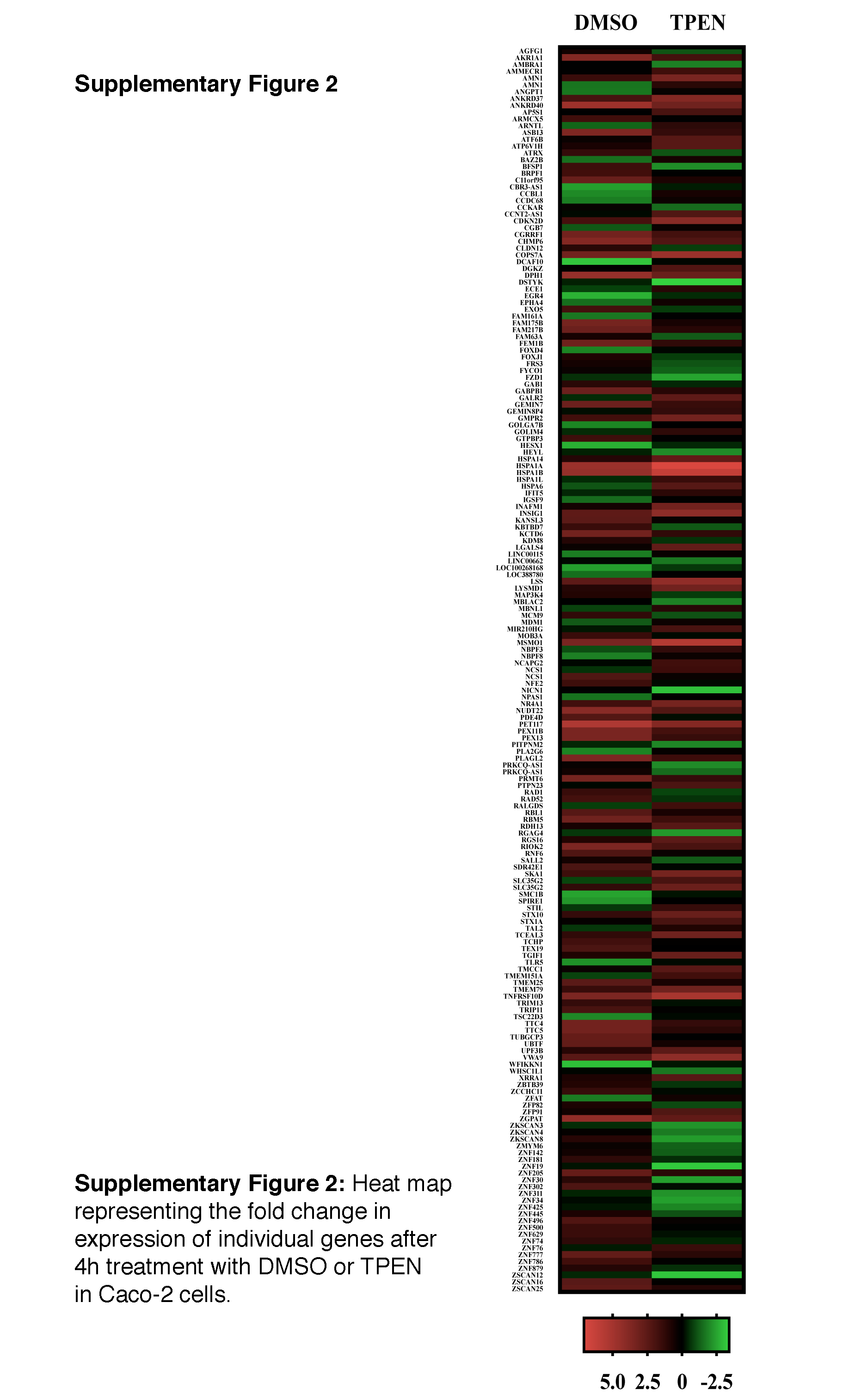

Supplement: Supplementary file 3 [file Image_2.TIF]
